# Redox DAPK1 destabilizes Pellino1 to govern inflammation-coupling tubular damage during septic AKI

**DOI:** 10.7150/thno.49870

**Published:** 2020-09-15

**Authors:** Bang-Chuan Hu, Guo-Hua Wu, Zi-Qiang Shao, Yang Zheng, Jin-Quan Liu, Run Zhang, Jun Hong, Xiang-Hong Yang, Ren-Hua Sun, Shi-Jing Mo

**Affiliations:** 1 Department of Intensive Care Unit, Zhejiang Provincial People's Hospital, People's Hospital of Hangzhou Medical College, Hangzhou 310014, Zhejiang, P.R.China.; 2 Zhejiang University School of Medicine, Zhejiang University, Hangzhou 310029, Zhejiang, P.R.China.

**Keywords:** Septic acute kidney injury, Tubular damage, DAPK1, Phosphorylation, Pellino1, Turnover

## Abstract

Tubular damage initiated by inflammatory response and ischemic/hypoxic stress is a hallmark of septic acute kidney injury (AKI), albeit the molecular mechanism coupling the two events remains unclear. We investigated the intrinsic nature of tubular damage with respect to inflammatory/hypoxic stress during septic AKI.

**Methods:** The apoptotic response of tubular cells to LPS stimuli was analyzed before and after hypoxia exposure. Cellular ubiquitination, co-immunoprecipitation, GST-pulldown, *in vitro* protein kinase assay, immunofluorescence and CRISPR technology were adopted to determine the molecular mechanism underlying this process. *In vivo* characterization was performed in wild-type and DAPK1^-/-^ mice models of cecal ligation and puncture (CLP).

**Results:** We found that the MyD88-dependent inflammatory response couples to tubular damage during LPS stimuli under hypoxia in a Fn14/SCF^Fbxw7α^-dispensable manner via recruitment of caspase-8 with TRIF-RIP1 signalosome mediated by DAPK1, which directly binds to and phosphorylates Pellino1 at Ser39, leading to Pellino1 poly-ubiquitination and turnover. Either pharmacological deactivation or genetic ablation of DAPK1 makes tubular cells refractory to the LPS-induced damage in the context of hypoxia, while kinase activity of DAPK1 is essential for ruin execution. Targeting DAPK1 effectively protects mice against septic AKI and potentiates the efficacy of a MyD88 homodimerization inhibitor, ST2825.

**Conclusion:** Our findings provide a rationale for the mechanism whereby inflammation intersects with hypoxic tubular damage during septic AKI through a previously unappreciated role of DAPK1-inducible Ser39 phosphorylation in Pellino1 turnover and underscore that combined targeting DAPK1 and MyD88 might be a feasible strategy for septic AKI management.

## Introduction

As one of the most troubled syndromes and common causes of death in intensive care unit (ICU), septic acute kidney injury (AKI) has been defined as a worldwide life-threatening disease, pathophysiologically characterized by tubular damage as a result of severe inflammatory response due to Gram-negative bacilli (e.g., *Pseudomonas aeruginosa and Acinetobacter baumannii*) infection and ischemic/hypoxic stress derived from septic shock 1, 2. To date, there is no satisfactory therapy in accelerating recovery from septic AKI. Herein, unveiling the precise mechanisms that govern pathogenesis of septic AKI would be helpful to develop innovative therapeutic strategies for this thorny disease.

The response of innate immune system to Gram-negative bacilli invasion during septic AKI relies on recognition of a limited but highly conserved set of molecular structures so-called pathogen-associated molecular patterns (PAMPs), including lipopolysaccharide (LPS), the main constituent of outer cell wall and virulence factor in Gram-negative bacilli 3. The pro-inflammatory nature of LPS primarily bases on its binding to toll-like receptor 4 (TLR4) and subsequent activation of myeloid differentiation primary response gene 88 (MyD88)/nuclear factor-κB (NF-κB) signaling pathway 4. Activated TLR4 upon LPS stimuli rapidly recruits the adapter protein MyD88, which cooperates with the interleukin-1 receptor-associated kinase 1 (IRAK1) and TNF receptor-associated factor 6 (TRAF6) to act on a series of intermediates for downstream signaling amplification, which phosphorylates IκB kinase β (IKKβ) and leads to NF-κB transactivation, thereby driving IL-6 transcription, production and sustaining inflammation 5.

Alternatively, recognition of LPS by TLR4 can sustain inflammatory responses by engaging another adapter protein, TIR domain-containing adapter-inducing interferon β (TRIF), that forms a macromolecular signalosome with receptor-interacting protein 1 (RIP1) through its homotypic interaction motif (RHIM). The TRIF-dependent cascade is thought to be competitive with the MyD88-dependent pathway, as TRIF signaling initiates after complex internalization into endosomes while MyD88 transduction results from TLR4/myeloid differentiation factor 2 (MD-2) complex located on cellular plasma membrane 6, 7. Moreover, RIP1 is dispensable for the MyD88-mediated NF-κB transactivation but has been implicated in heterogenous regulation of cell fate following TLR activation 8. It is noteworthy that the macromolecular TRIF-RIP1 signalosome recruits E3 ubiquitin ligase Pellino1, which renders the K63-linked ubiquitination of the cellular inhibitor of apoptosis protein 2 (cIAP2) to prevent caspases activation and maintain cell survival 9, 10. Pellino-1 also overcomes cytotoxicity through upregulation of cIAP1/2 expression 11.

In addition to evoke inflammatory responses, LPS can also orchestrate cell death in a context-dependent manner. Our recent study demonstrated that LPS stimuli triggers tubular cell apoptosis under nutrient-poor conditions. The disassembly of Fn14 from E3 ligase SCF^Fbxw7^ is sufficient to dismantle the K48-linked polyubiquitination of Fn14 and stabilizes it, while pharmacological deactivation of Fn14 effectively prevents the LPS-stimulated tubular damage *in vitro* and provides kidney protection against septic AKI in mice 12. On the other hand, deregulated redox homeostasis appears to play a pivotal role in sepsis. Redox factors affecting septic pathology have also been identified. For instance, increased nitric oxide levels and changes in reactive oxygen species (ROS) production elicited by hypoxia suppresses mitochondrial respiration and contributes to sepsis-associated organ failure (OF) 13, 14. The connection of redox factors with sepsis-associated OF raises the possibility that hypoxic stress might function as a manipulator of inflammation-related tissue injury. In a process that is still not fully understood so far, the pro-apoptotic behavior of LPS is observed when cells are costimulated with hypoxia 15-17. Recently, the contribution of TRIF and RIP1 to LPS-dependent cell apoptosis has been documented in emerging studies. That is, the LPS-dependent cell apoptosis is mediated by TRIF and RIP1 interaction which results in caspase-8 activation 18. However, the precise mechanisms of how LPS orchestrates tubular apoptosis under hypoxia remain unclear and little is known about the intrinsic nature of inflammatory/hypoxic tubular damage during septic AKI.

Death-associated protein kinase 1 (DAPK1) is a calcium/calmodulin-regulated serine/threonine kinase which, once activated, induces cell apoptosis through phosphorylating downstream substrates in response to hypoxic stress 19, 20. Dephosphorylation of amino acid (aa), specifically serine (Ser)308 residue, activates DAPK1 and control its catalytic activity 21. Under hypoxic stress conditions, DAPK1 phosphorylates numerous downstream substrates through the preferential consensus motif R-R-*x-*S* (*x* represents any aa and *denotes the phosphorylated residue) 22. Meanwhile, DAPK1 is able to phosphorylates N-myc downstream-regulated gene 2 (NDRG2) at Ser350 residue for apoptotic induction 23. Intriguingly, DAPK1 has been found to facilitate release of IL-1β, the pro-inflammatory cytokine whose catalytic activity is tightly controlled by caspase-1 following activation of pattern recognition receptors (PRRs) 24. DAPK1 is also identified as a key factor to promote development and progression of acute respiratory distress syndrome (ARDS), where TLR4-mediated inflammatory cells infiltration and ischemic/hypoxic injury represent the common features 25. Nevertheless, the exact mechanism concerning the reciprocity between DAPK1 and TLR4 signaling is undefined and the significance of DAPK1 in septic AKI has not been comprehensively studied.

In the present study, we uncover that the MyD88-dependent inflammatory response couples to tubular damage during LPS stimuli under hypoxia in a Fn14/SCF^Fbxw7α^-dispensable fashion through releasing TRIF-RIP1 signalosome for caspase-8 recruitment mediated by DAPK1, which directly binds to and phosphorylates Pellino1 at Ser39, leading to Pellino1 polyubiquitylation and degradation. These findings reinforce the notion that hypoxia empowers LPS to sustain inflammation and simultaneously induce tubular damage through a distinguishable Pellino1 Ser39 phosphorylation and turnover triggered by DAPK1. Our study also identifies DAPK1 as a putative therapeutic target for septic AKI and demonstrates that pharmacological deactivation or genetic ablation of DAPK1 in combination with MyD88 inhibitor provides better efficacy against septic AKI.

## Materials and Methods

### Reagents and antibodies

The *Escherichia coli* 0111: B4 LPS, *N*-hexanoyl-D-sphingosine (C6-ceramide) and zVAD-FMK were obtained from Sigma-Aldrich (St. Louis, MO, USA). DAPK1 inhibitor and ST2825 were purchased from Medchem Express (Monmouth Junction, New Jersey, USA). Recombinant GST-DAPK1 fusion protein was obtained from Millipore (Billerica, MA). Caspase-3 activity detection kit was from Bestbio (Shanghai, China). Quantikine human IL-6 ELISA kit was from R&D Systems (Minneapolis, MN). Annexin V-FITC apoptosis detection kit was ordered from Beyotime (Nanjing, China). The following antibodies with the company and concentration were used for coimmunoprecipitation (co-IP) or western-blotting analyses: anti-Pellino1 (Abcam, 1:500), anti-MyD88 (Cell Signaling Technology, 1:500), anti-caspase-8 (Cell Signaling Technology, 1:500), anti-TRIF (Cell Signaling Technology, 1:1000), anti-RIP1 (BD Biosciences, 1:2000), anti-Flag (ProteinTech group, 1:1000), anti-phospho-DAPK1 (Sigma-Aldrich, 1:1000), anti-DAPK1 (Cell Signaling Technology, 1:1000), anti-Fbxw7 (Abcam, 1:1000), anti-pSer (Santa Cruz, 1:1000), anti-Fn14 (Cell Signaling Technology, 1:2000) and anti-GAPDH (Biosynthesis, 1:3000).

### Plasmids and CRISPR-Cas9 gene editing

His-tagged Ub and Fn14 siRNA were described in previous publications 12. Human Pellino1 cDNA was amplified by PCR and subcloned into pMSCV-Flag retroviral vector (Addgene, Cambridge, MA). Pellino1 S39A and S39D mutants were constructed using QuickChange^®^ Site-Directed Mutagenesis Kit (Agilent Technologies, Santa Clara, CA) with the primers as below: S39A sense, 5′-CGATAGAGGAAGGAGGAAAGCCAGGTTTGCTTTGTTTAAAAG-3′ and S39A antisense, 5′-CTTTTAAACAAAGCAAACCTGGCTTTCCTCCTTCCTCTATCG-3′; S39D sense, 5′-GCGATAGAGGAAGGAGGAAAGACAGGTTTGCTTTGTTTAAAAG-3′; and S39D antisense, 5′-CTTTTAAACAAAGCAAACCTGTCTTTCCTCCTTCCTCTATCGC-3′. The V5-tagged DAPK1 was constructed using pLX304 vector (Genecopoeia, Rockville, MD). The kinase-dead (KD) DAPK1 mutant was generated as described previously 26. RIP1 and RIP3 siRNA were purchased from Dharmacon (Lafayette, CO). Caspase-8 siRNA and DAPK1 shRNA were ordered from Santa Cruz Biotechnology (Santa Cruz, CA). For CRISPR-Cas9 gene knockout, CRISPR guide sgRNAs targeting Pellino1 and DAPK1 were subcloned to commercial pCRISPR-SG01 vectors (HCP254204-SG01-3 and HCP270000-SG01-3) which were purchased from GeneCopoeia (Rockville, USA).

### Cell culture and transfection

The culture protocols for murine macrophage-like RAW 264.7 and tubular epithelial MCT cells were described in previous studies 12. Human HK-2 cells from ATCC (American Type Culture Collection) were maintained in Dulbecco's modified Eagle's medium (Gibco, Carlsbad, USA) containing 10% FBS, 4 mM L-glutamine, 100 IU penicillin and 100 mg/mL streptomycin under humidified atmosphere at 37 °C in 5% CO_2_ and 95% air. Hypoxic preconditioning of the LPS-primed cells was performed as described previously 27. Procedures for transient and stable transfection had been reported previously 12, 27, 28. Transient siRNA transfection was carried out using Lipofectamine 2000 reagent (Invitrogen, Carlsbad, CA) according to manufacturer's instructions. For shRNA or plasmid DNA transfection, the stable transfectants were isolated, pooled and expanded for further analysis beginning 48 h after ten days of puromycin (0.5 μg/mL) or two weeks of blasticidin (10 μg/mL) selection. Pellino1 knockout cell lines with CRISPR-Cas9 gene editing were selected by hygromycin (250 μg/mL) and the knockout efficacy was evaluated using western blotting.

### Cellular ubiquitination and co-immunoprecipitation assay

The detailed guidelines for cellular ubiquitination and co-immunoprecipitation were described previously 12, 27, 28. For the cellular ubiquitination assay, HK-2 cells transfected with the indicated plasmids were underwent variable treatment and lysed with the denatured buffer (6 M guanidine-HCl, 0.1 M Na_2_HPO_4_/NaH_2_PO_4_, 10 mM imidazole).The cell lysates were then incubated with Ni^2+^-nitrilotriacetic acid (NTA)-sepharose beads for 3 h, washed, and subjected to western blotting analysis. For co-immunoprecipitation assay, cells were washed with 1×PBS and then solubilized on ice in radio-immunoprecipitation assay (RIPA) buffer (KeyGen BioTech, Nanjing, China) containing 50 mM Tris, 150 mM NaCl, 1% NP-40, 0.25% sodium deoxycholate and protease inhibitors. After centrifugation at 14,000 rpm for 10 min at 4 °C, the supernatants were transferred to the fresh tubes and then incubated with primary antibodies at 4 °C followed by a further rotation with protein A/G- agarose beads (Cwbiotech, Beijing, China) overnight. After rinsing three times with the lysis buffer, immunoprecipitated proteins were boiled for 10 min in sample buffer and analyzed by western blotting.

### GST-pulldown assay

Immunoprecipitates of Flag from HK-2 cell lysates expressing Flag-tagged wild-type Pellino1 were mixed with GST or GST-DAPK1 fusion protein for 30 min at 30 °C, followed by incubation with Glutathione beads (Sigma-Aldrich, St.Louis, MO, USA) for 2 h. Reaction products were subjected to sodium dodecyl sulfate-polyacrylimide gel electrophoresis (SDS-PAGE) and blotted with the indicated antibodies.

### *In vitro* protein kinase assay

Cells were transfected with constructs expressing Flag-tagged wild-type Pellino1 or Pellino1 S39A mutant. Wild-type or mutant Pellino1 was purified and incubated with 1 µg of recombinant GST-DAPK1 fusion protein in the presence of 0.2 mM ATP for 30 min. Reaction was terminated by addition of SDS-containing lysis buffer and the reaction products were resolved by SDS-PAGE and detected by western-blotting.

### Immunofluorescence

Immunofluorescence staining was conducted as previously described with a few modifications 27. Briefly, cells cultured in six-well were fixed with 4% formaldehyde in PBS for 10 min at 37 °C. After permeabilizing with 1% Triton X-100 for 10 min, they were blocked with 5% BSA in PBS and Tween-20 (PBST) at 37 °C for 1h and incubated with specific antibody against DAPK1 (1:250) and Flag (1:250) overnight. The cells were then incubated with secondary anti-rabbit antibody conjugated with Alexa Fluor 594 (1:500) at 37 °C for 1 h. After being washed with 1×PBS for three times, cells were stained by 5 µg/mL 4', 6-diamidino-2-phenylindole (DAPI) in PBS for 15 min. Immunofluorescence images were acquired on a fluorescent microscope (IX71; Olympus, Japan).

### Apoptosis measurement

#### Cell viability assay

Cell viability was determined by 3-(4,5-dimethylthiazol-2-yl)-2,5-diphenyltetra-zolium bromide reduction (MTT) assay as indicated previously 27, 29. In brief, the indicated cells were seeded in 96-well plates at a density of 1×10^4^ per well and underwent various treatment with each incubation time point except on day zero. Before the end of the experiment, 20 μL MTT (5 mg/mL; Sigma-Aldrich) was added and the plates were incubated at 37 °C for further 4h. Subsequently, 150 mL dimethyl sulfoxide (DMSO) was added to dissolve formazan and the absorbance was measured at 570 nm by spectrometer (Wellscan MK3; Labsystems Dragon).

#### Flow cytometry

Flow cytometry with Annexin-V/Propidium iodide (PI) double staining was performed to determine cell apoptosis as described in previous publications 12, 29. Briefly, the indicated cells were seeded in 24-well plates, then harvested, washed and incubated with binding buffer containing 25 μg/mL Annexin V-FITC and 25 μg/mL PI at 37 °C. Twenty minutes later, the cells suspensions were subjected to FACS analysis.

#### Hoechst-PI staining

The indicated cells were grown in six-well plates and washed twice with 1× PBS. Hoechst 33342, a blue-fluorescent dye that stains all cells and propidiumiodide (PI), a red-fluorescent dye that stains dead cells, were subsequently added to culture medium at a final concentration of 5 µg/mL. After incubation for 15 minutes, quantification of apoptotic cells were observed and quantified using fluorescent microscope (IX71; Olympus, Japan) as previously described 28, 29. The percentage of apoptotic cells were expressed as the mean ± standard deviation of three independent experiments.

### DEVDase activity assay

DEVDase activity assay was carried out to assess Caspase-3 activation as described previously 12, 29. In brief, cellular proteins from different groups were extracted and their concentrations were measured using the Bradford protein assay. The protein liquids were then mixed with the reaction buffer and 10 μL Ac-DEVD-*p*NA substrate, followed by 1 h incubation at 37 °C. DEVDase activity was measured at 405 nm with a plate reader as recommended in the manufacturer's instructions. Three independent experiments were performed separately.

### Enzyme-linked immunosorbent assay (ELISA)

Interleukin-6 (IL-6) secretion in cell culture medium was determined by ELISA using the Quantikine human IL-6 ELISA kit according to the manufacturer's guidelines. The absorbance of the samples was measured by Synergy H4 Hybrid Reader (Biotek, Winooski, VT) with wavelength set at 450 nm. The assay was performed in triplicate and standard deviation representing experimental errors were calculated.

### RNA extraction and real-time quantitative PCR (RT-qPCR)

The detailed procedures for RT-qPCR were as described previously 12, 29. In general, complementary DNA was synthesized from total RNA with Super Array PCR master mix (SuperArray Bioscience, USA) using oligo(dT) as a primer. Gene transcripts were quantified by real-time PCR using Takana SYBR^®^ Primix Ex Taq^TM^Kit (Takana, China) on an Applied Biosystems 7900HT cycler. All of the values of the target gene expression level were normalized to GAPDH. The following primers were used in real-time PCR: IL-6: 5′-ACTCACCTCTTCAGAACGAATTG-3′ (forward), 5′-CCATCTTTGGAAGGTTCAGGTTG-3′ (reverse); PELI1: 5′-CAGCACTGTGCATATTGCTTG-3′ (forward), 5′-CGGCCAATCTGAAACATATCGG-3′ (reverse); GAPDH: 5′-AATCCCATCACCATCTTCC-3′ (forward), GAPDH: 5′-TGGACTCCACGACGTACTC-3′ (reverse).

### Western-blotting

Western blotting analyses were performed with precast gradient gels (Bio-Rad) using standard methods as described previously 12, 27. Briefly, cells were pelleted by centrifugation and resuspended in 0.5 mL ice-cold RIPA buffer containing protease inhibitors and phosphatase inhibitors by pipetting up and down about 10 times. After incubation on ice for 10 minutes, the lysates were centrifuged at 15,000 g and the supernatants were then transferred to another fresh tube. Proteins were fractionated on 10% SDS-PAGE and transferred to the Immobilon™ PVDF Transfer Membranes (Millipore Corporation, Billerica, MA). After blocked in 5% bovine serum albumin (BSA), the membrane was incubated with the indicated primary antibodies overnight and then with HRP-conjugated secondary antibody (Biosythesis, Beijing, China). The bands were visualized by western chemiluminscent HRP substrate kit (PPLYGEN, Beijing, China).

### Animal study

The DAPK1^-/-^ and DAPK1^+/+^ (wild-type) C57BL/6 J background mice were purchased from Shanghai SLAC Laboratory Animal Co., Ltd (Shanghai, China) and bred in the specific-pathogen-free animal facility of the Animal Experiment Center of Zhejiang University. Cecal Ligation and Puncture (CLP) experiments were performed as previously described with some modifications 12. Briefly, after being anesthetized with ketamine (100 mg/kg), a midline abdominal incision was made, and the cecum was exteriorized and ligated with 4-0 silk immediately distal to the ileocecal valve without causing intestinal obstruction. The cecum was then punctured twice with a 22-gauge needle and placed back into its normal intraabdominal position. The abdomen was closed with a running suture of prolene in two layers to prevent leakage of fluid. DAPK1 inhibitor (1 mg/kg) and ST2825 (0.2 mg) or both were administered intraperitoneally (i.p.) to mice at 24, 48 and 72 hours after CLP with the indicated doses, respectively. Serum samples was collected and stored at -20 °C before analysis. Mortality was recorded for more than one week after the onset of CLP to ensure that no additional late deaths occurred. All animal studies were conducted with the approval of the Zhejiang University Institutional Animal Care and Use Committee and were performed in accordance with established guidelines.

### H&E and TUNEL staining

For H&E staining, dissected kidney tissues were cut into sagittal blocks and fixed with 4% paraformaldehyde, followed by dehydration and paraffin embedding. Sections 5μm in thickness were cut from the paraffin-embedded tissue blocks with a Leica slicing machine and mounted on poly-D-lysine coated glass slices. Slices were heated at 65 °C for 2 h and then immersed in xylene to remove paraffin. After a series of rehydration processes, slices were stained with hematoxylin and eosin (HE) and imaged with the AxioVision Rel.4.6 computerized image analysis system (Carl Zeiss). TUNEL assay was conducted on the indicated paraffin-embedded kidney tissue blocks using an *In situ* Cell Death Detection Kit (Roche Inc., Indianapolis, USA) just as the given protocol.

### Immunochemical staining

Immunohistochemical staining was carried out as described in previous publications 12. In brief, sections cut were deparaffinized and rehydrated with serial passage through changes of xylene and graded alcohol. After antigen retrieval using either citrate or EDTA buffer, endogenous peroxidase in tissues was blocked by incubation of slides in 2% hydrogen peroxide solution before incubation with primary antibody. Images were obtained using standard methods and imaged with a AxioVision Rel.4.6 computerized image analysis system (Carl Zeiss). At least three repeats were conducted for each calculation.

### Statistical analysis

Statistical analyses were performed using SPSS 17.0 software (SPSS Inc., Chicago, IL, USA). An unpaired, two-tailed Student's *t* test was used to determine differences between two groups and statistical comparisons in multigroup analysis were assessed by one-way ANOVA. Data were expressed as mean ± standard deviation of at least three independent experiments and *P* value of < 0.05 was considered statistically significant.

## Results

### MyD88-dependent inflammatory response stimulated by LPS couples to tubular damage without affecting Fn14/SCF^Fbxw7α^ cascade under hypoxia

**Figure 1:**
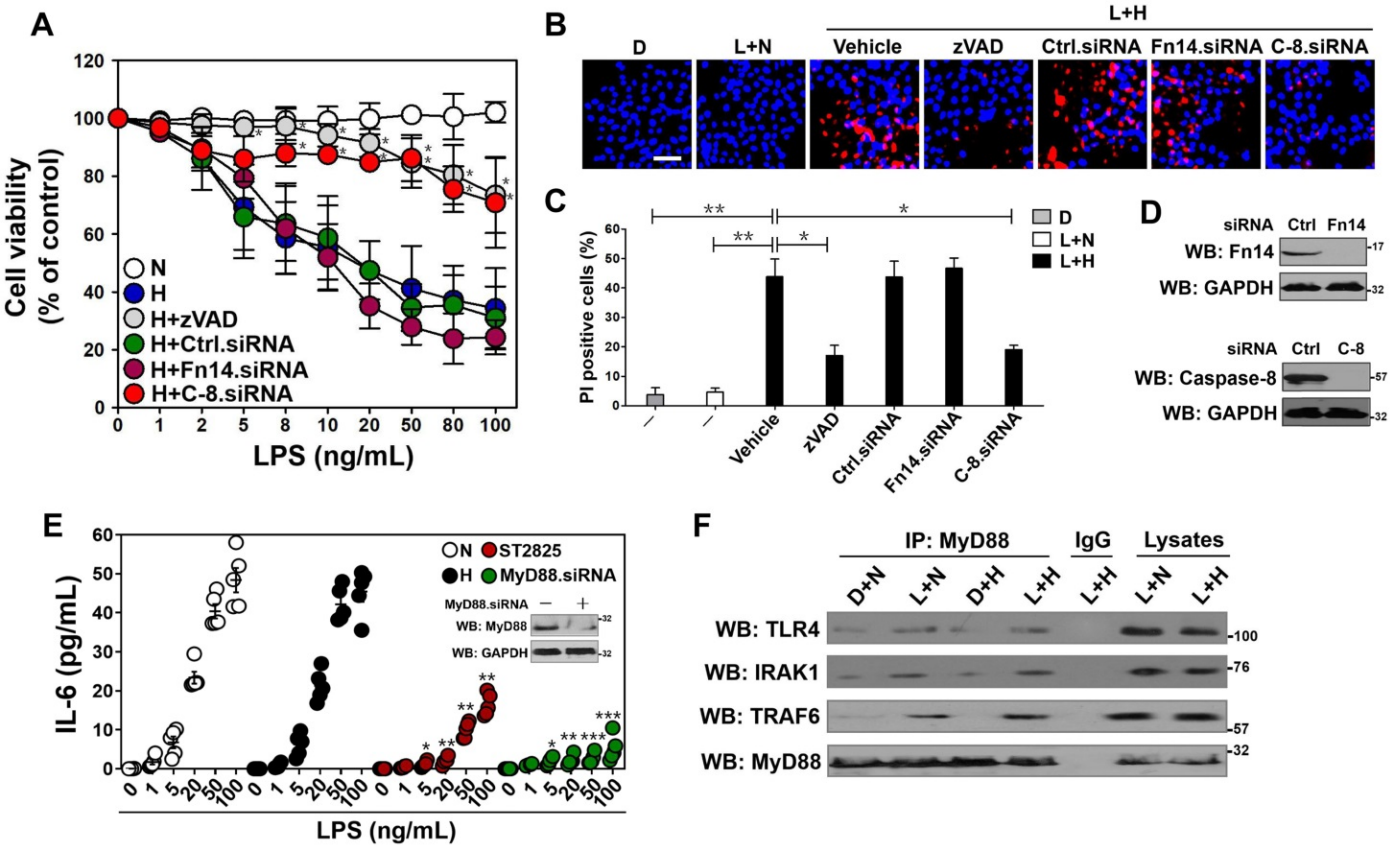
** MyD88-dependent inflammatory response stimulated by LPS couples to tubular damage without affecting Fn14/SCF^Fbxw7α^ cascade under hypoxia.** (A) MTT assay measuring cell viability of HK-2 cells exposed to LPS at the indicated concentrations with or without hypoxia in the presence of 20 µM zVAD-FMK and siRNA targeting control, Fn14 or caspase-8 transfection, respectively. N: normoxia. H: hypoxia. Ctrl.siRNA: control siRNA. C-8.siRNA: caspase-8 siRNA. Experiments were performed three times and data are expressed as mean ± s.d. *P<0.05 versus H, one-way ANOVA, post hoc comparisons, Tukey's test. (B and C) Representative pictures (B) and quantification (C) from Hoechst33342 and PI double-staining assay of HK-2 cells exposed to 50 ng/mL LPS with or without hypoxia in the presence of 20µM zVAD-FMK and siRNA targeting Fn14 or caspase-8 transfection, respectively. D: DMSO, L: LPS. Data are expressed as mean ± s.d. of three independent experiments. *P<0.05, **P<0.01, one-way ANOVA, post hoc comparisons, Tukey's test. (D) Western blotting analyses detecting the abundance of Fn14 (top panel) and caspase-8 (bottom panel) protein in HK-2 cells with Fn14 or caspase-8 siRNA transfection. (E) ELISA assay for IL-6 secretion from HK-2 cell cultures exposed to LPS at the indicated concentrations with hypoxia or 10 µM ST2825 treatment or MyD88 siRNA transfection. Data are expressed as mean ± s.d. of at least three experiments. *P<0.05, **P<0.01, ***P<0.001 versus N, one-way ANOVA, post hoc comparisons, Tukey's test. Insert: Western blotting analyses comparing the levels of MyD88 protein in HK-2 cells with or without MyD88 siRNA transfection. (F) Coimmunoprecipitation assay evaluating the interaction between MyD88 and TLR4, IRAK1 and TRAF6 in LPS-stimulated HK-2 cells with or without hypoxia. IP: immunoprecipitation.

LPS has been reported to sensitize cells to death under certain circumstance such as hypoxia 17. To further corroborate and extend this finding, we stimulated human kidney proximal tubular epithelial HK-2 cells with LPS at different concentrations ranging from 0 to 100 ng/mL, followed by incubating them under normoxic or hypoxic conditions for 24 h. Cell viability curves, as measured by MTT assay, showed that LPS by itself had minimal effects on survival of HK-2 cells at any dose after stimuli but decreased their survival under hypoxia (*P<* 0.01, Figure 1A, 50 ng/mL LPS was applied in subsequent experiments because the maximal rescue was observed by both caspase inhibition and caspase-8 depletion at this concentration [see below]). As well, data from flow cytometry (FCM) with Annexin-V/PI staining demonstrated that LPS plus hypoxia costimuli increased the proportion of apoptotic cells by average 50%, whereas either LPS or hypoxia single stimuli failed to do so (*P <* 0.01, Figure S1A). The MTT and FCM results were consistent with Hoechst/PI analyses showing that the percentage of PI-positive apoptotic staining in cell cultures costimulated with LPS plus hypoxia were much higher than those in cell cultures that were stimulated with LPS alone (*P <* 0.01, Figure 1B-C). Treatment with cobalt chloride (CoCl_2_), a widely utilized hypoxia-mimetic reagent, killed cells in a LPS dose-dependent fashion as effectively as hypoxia did (*P <* 0.05 and *P <* 0.01, Figure S1B). In contrast to LPS single stimuli, LPS plus hypoxia costimuli activated caspase-3, as evidenced by the appearance of increased DEVDase (an indicative of caspase-3 enzymatic activity 30) amounts over doses (*P <* 0.05, Figure S1C). Notably and in accordance with the data described for higher DEVDase activities upon LPS plus hypoxia costimuli, pretreatment with benzyloxycarbonyl-Val-Ala-Asp-fluoromethylketone (zVAD-FMK), a pan caspase inhibitor, abrogated the diminution of survival (*P <* 0.05, Figure 1A) and the elevation of PI-positive apoptotic staining (*P <* 0.05, Figure 1B-C) in the LPS plus hypoxia-costimulated HK-2 cells. The similar phenomenon was recapitulated in murine macrophage-like RAW264.7 and tubular epithelial MCT cells (*P <* 0.05, Figure S1D). These data together suggest that hypoxia empowers LPS to induce caspase-dependent tubular cell apoptosis.

Given our previous study demonstrated that Fn14 disassociates from SCF^Fbxw7α^ and contributes to the LPS-induced tubular apoptosis 12, we reasoned that Fn14 is involved in the apoptotic phenotypes caused by LPS plus hypoxia costimuli. To approach this, we depleted Fn14 using small interfering RNA (siRNA) (Figure 1D, top panel) and costimulated the Fn14-depleted HK-2 cells with LPS and hypoxia. Unexpectedly, the LPS plus hypoxia-costimulated cells underwent apoptosis regardless of Fn14 depletion (Figure 1A-C). Meanwhile, negligible effect of hypoxia on the LPS-stimulated disassociation of Fn14 from SCF^Fbxw7α^ was observed (Figure S1E). In sharp contrast, the siRNA-mediated ablation of caspase-8 (the executioner for TLR-initiated cell death 31) eventually mitigated apoptosis of HK-2 cells under the same costimuli conditions (*P <* 0.05, Figure 1A-D), suggesting that caspase-8 contributes to the LPS plus hypoxia-induced apoptosis of HK-2 cells independent of Fn14/SCF^Fbxw7α^ cascade. These data collectively indicate that Fn14/SCF^Fbxw7α^ cascade might be not the main mechanism whereby LPS induces the caspase-8-dependent tubular damage in response to hypoxia.

Since MyD88/NF-κB/IL-6 cascade is the downstream effector responsible for TLR4 activaton 32, we tested whether it participates in the Fn14/SCF^Fbxw7α^-independent, caspase-8-dependent tubular apoptosis induced by LPS under hypoxia. To this end, we measured mRNA expression and secretion of IL-6 using real-time quantitative reverse transcriptase-polymerase chain reaction (RT-qPCR) analyses and enzyme-linked immunosorbent assay (ELISA) assay, respectively. Unexpectedly, no significant differences of IL-6 mRNA expression and protein secretion were observed in the LPS-stimulated HK-2 cells before and after hypoxia exposure (Figure S1F, Figure 1E). However, LPS single stimuli could no longer enhance IL-6 secretion in cells with ST2825 (a selective inhibitor of MyD88 homodimerization) pretreatment or MyD88 siRNA transfection to the same extent as it did in cells with or without hypoxia exposure (*P <* 0.05, *P <* 0.01 and *P <* 0.001, Figure 1E), indicating that hypoxia cooperates with LPS to trigger tubular apoptosis but does not influence the LPS-induced inflammatory response. In echoing this notion, the LPS plus hypoxia-costimulated cells had almost equivalent levels of p-IKKβ and total IκBα to the LPS-stimulated cells (Figure S1G). Western-blotting of immunoprecipitated MyD88 with antibodies against TLR4, IRAK1 and TRAF6 showed that the LPS-stimulated interaction of MyD88 with TLR4, IRAK1 and TRAF6 at endogenous levels was barely changed following hypoxia exposure (Figure 1F). Taken together, these data suggest that the MyD88-dependent inflammatory response couples to the Fn14/SCF^Fbxw7α^-independent, caspase-8-dependent tubular apoptosis after LPS stimuli under hypoxic conditions.

### Hypoxia destabilizes Pellino1, releasing TRIF-RIP1 signalosome to recruit caspase-8 and inducing tubular damage upon LPS stimuli

**Figure 2:**
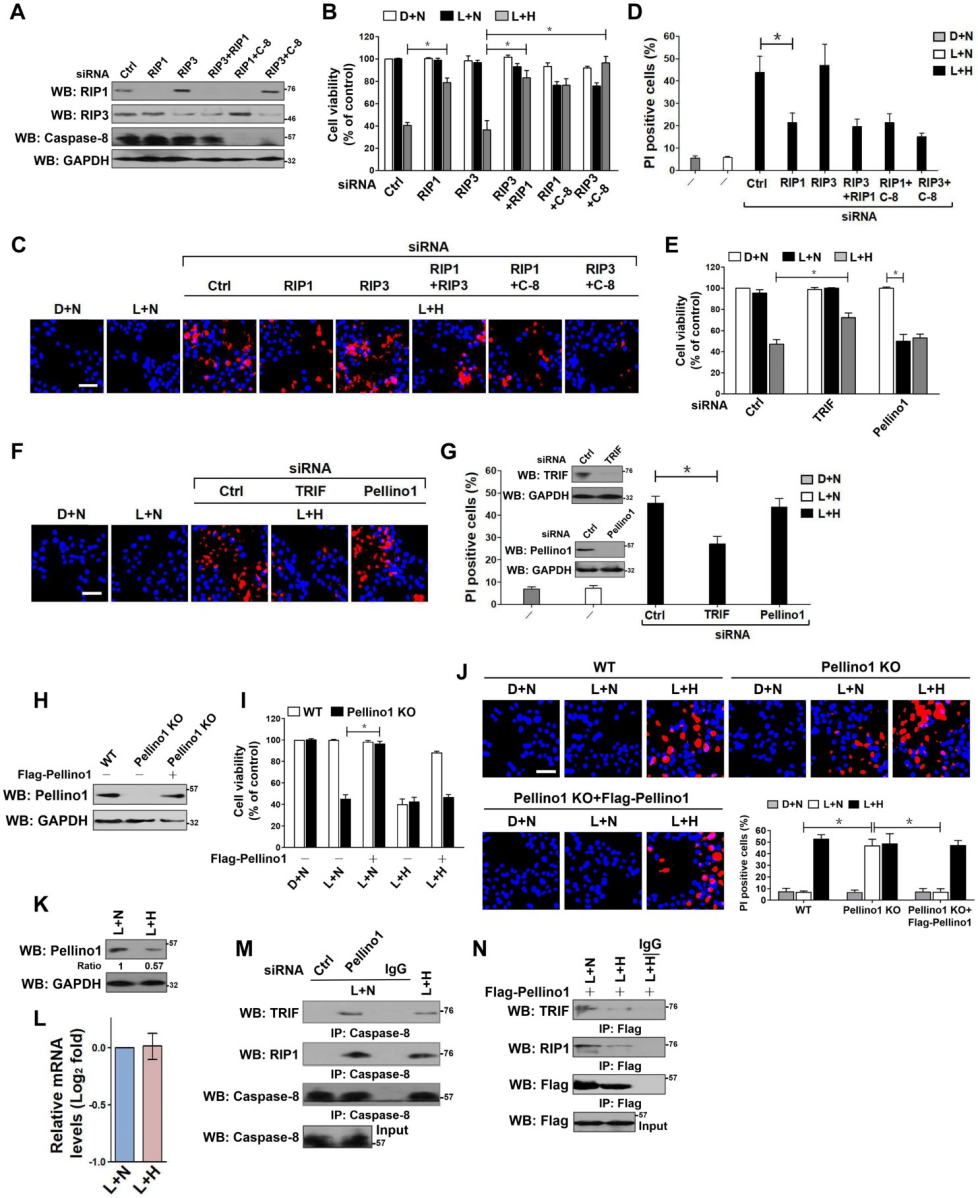
** Hypoxia releases TRIF-RIP1 signalosome to recruit caspase-8 and induces tubular damage upon LPS stimuli in a Pellino1-dependent manner.** (A) Western blotting analyses comparing levels of RIP1, RIP3 or caspase-8 protein expression in HK-2 cells transfected with siRNA targeting RIP1, RIP3 and caspase-8 or both of each other. (B) MTT assay comparing cell viability of HK-2 cells exposed to 50 ng/mL LPS with or without hypoxia in the presence of siRNA targeting RIP1, RIP3, caspase-8 or both of each other. Experiments were performed three times and data are expressed as mean ± s.d. *P<0.05, one-way ANOVA, post hoc comparisons, Tukey's test. (C and D) Representative pictures (C) and quantification (D) from Hoechst33342 and PI double-staining assay of HK-2 cells exposed to 50 ng/mL LPS with or without hypoxia in the presence of siRNA targeting RIP1, RIP3 and caspase-8 or both of each other. Experiments were performed three times and data are expressed as mean ± s.d. *P<0.05, one-way ANOVA, post hoc comparisons, Tukey's test. (E) MTT assay measuring cell viability of HK-2 cells exposed to 50 ng/mL LPS with or without hypoxia in the presence of siRNA targeting TRIF or Pellino1. Experiments were performed three times and data are expressed as mean ± s.d. *P<0.05, one-way ANOVA, post hoc comparisons, Tukey's test. (F and G) Representative pictures (F) and quantification (G) from Hoechst33342 and PI double-staining assay of HK-2 cells exposed to 50 ng/mL LPS with or without hypoxia in the presence of siRNA targeting TRIF or Pellino1. Experiments were performed three times and data are expressed as mean ± s.d. *P<0.05, one-way ANOVA, post hoc comparisons, Tukey's test. Insert: Western blotting analyses detecting expression of TRIF or Pellino1 protein in HK-2 cells transfected with siRNA targeting TRIF or Pellino1. (H) Western blotting analyses detecting levels of Pellino1 protein in Pellino1 knockout HK-2 cells (Pellino1 KO) where Pellino1 was deleted from the genome by CRISPR-Cas9 editing in the presence or absence of Flag-tagged wild-type Pellino1 expression. (I) MTT assay examining cell viability of Pellino1 knockout HK-2 cells exposed to 50 ng/mL LPS with or without hypoxia in the presence or absence of Flag-tagged wild-type Pellino1 expression. Experiments were performed three times and data are expressed as mean ± s.d. *P<0.05, one-way ANOVA, post hoc comparisons, Tukey's test. (J) Representative pictures and quantification from Hoechst33342 and PI double-staining assay of Pellino1 knockout HK-2 cells exposed to 50 ng/mL LPS with or without hypoxia in the presence or absence of Flag-tagged wild-type Pellino1 expression. Experiments were performed three times and data are expressed as mean ± s.d. *P<0.05, one-way ANOVA, post hoc comparisons, Tukey's test. (K and L) Western blotting and RT-qPCR analyses assessing levels of Pellino1 protein (K) and mRNA (L) expression in HK-2 cells exposed to 50 ng/mL LPS with or without hypoxia exposure. (M) Coimmunoprecipitation assay examining the interaction of caspase-8 with TRIF and RIP1 in LPS-stimulated HK-2 cells with Pellino1 siRNA transfection or with hypoxia exposure. (N) Coimmunoprecipitation assay examining the interaction of Flag-Pellino1 with TRIF and RIP1 in LPS-stimulated HK-2 cells with or without hypoxia exposure.

Both RIP1 and RIP3 have been demonstrated to be responsible for the LPS-dependent cell death 33. We thus asked whether RIP1 or RIP3 is essential for the observed apoptosis in HK-2 cells. To this end, we depleted RIP1, RIP3 and both in HK-2 cells using RIP1 and/or RIP3 RNAi, and then costimulated them with LPS plus hypoxia. Depletion of RIP1, but not that of RIP3, blocked the reduction in cell survival and the elevation in PI-postitive apoptosis staining induced by LPS plus hypoxia (*P <* 0.05, Figure 2A-D). Although the RIP3-depleted cells were killed to a similar extent as the control cells upon LPS plus hypoxia costimuli, this phenotype could be rescued by cotransfecting with RIP1 or caspase-8 RNAi (*P <* 0.05). Double depletion of RIP1 and caspase-8 showed no additive effects in restoring cellular survival, suggesting that RIP1 and caspase-8 might act in the same pathway to participate in the LPS-dependent apoptosis under hypoxia. The roles of other two LPS-responsive signaling molecules, TRIF and Pellino1, in the LPS-dependent apoptosis under hypoxia were also investigated. The RNAi-mediated silencing of TRIF, rather than Pellino1, counteracted HK-2 cell apoptosis induced by LPS plus hypoxia (*P <* 0.05, Figure 2E-G). We noticed that stimulation of the Pellino1-silenced cells with LPS greatly decreased their survival even in the absence of hypoxia (*P <* 0.05, Figure 2E), suggesting that Pellino1 deficiency is sufficient to mimicking hypoxia for apoptotic induction upon LPS stimuli. Indeed, knockout of Pellino1 using CRISPR-Cas9 genome editing in HK-2 cells increased their apoptosis after LPS stimuli, whereas restoration of Pellino1 expression using a Flag-tagged wild-type Pellino1 eliminated this effect (*P <* 0.05, Figure 2H-J). Depletion of caspase-8, but not Fn14, attenuated the LPS-dependent apoptosis in Pellino1-knockout HK-2 cells (*P <* 0.05, Figure S2A-D). However, Pellino1 knockout did not significantly change the physical interaction of MyD88 with TLR4, IRAK1 and TRAF6 stimulated by LPS. To decipher whether hypoxia facilitates the LPS-dependent cell apoptosis through repressing Pellino1, we compared the levels of Pellino1 protein and mRNA in the LPS-stimulated HK-2 cells with or without hypoxia exposure. As shown in Figure 2K-L, hypoxia appreciably reduced the abundance of Pellino1 protein yet had no effect on its mRNA expression. These data indicate that TRIF, RIP1 and caspase-8, are engaged in the LPS-dependent tubular cell apoptosis under hypoxia, for which Pellino1 protein degradation might be required.

Coincide with the engagement of TRIF, RIP1 and caspase-8 in the LPS-dependent apoptosis under hypoxia, both TRIF and RIP1 were detected in the immunoprecipitates (IPs) of caspase-8 from the LPS plus hypoxia-costimulated HK-2 cells (Figure S2E). The similar phenomenon was observed in the Pellino1-silencing cells with LPS single stimuli (Figure 2M), indicating that LPS is able to induce cell apoptosis in the case of hypoxia or Pellino1 deficiency via evoking TRIF and RIP1 to recruit caspase-8. Based on the aforementioned data showing that hypoxia reduced the levels of Pellino1 protein, we attempted to clarify whether hypoxia releases TRIF and RIP1 for caspase-8 recruitment through destabilizing Pellino1 during LPS stimuli. For this purpose, we transfected the LPS plus hypoxia-costimulated HK-2 cells with Flag-Pellino1 and performed western-blotting (WB) on the IPs of Flag using anti-TRIF and anti-RIP1 antibodies individually. Figure 2N showed that hypoxia diminished the amount of TRIF and RIP1 in IPs of Flag-Pellino1 from the LPS-stimulated cells during the tested time frame, probably owing to the increased Pellino1 protein degradation. Reciprocally, WB analyses of immunoprecipitated RIP1 with antibodies against Flag, TRIF and caspase-8 depicted that hypoxia disrupted the LPS-stimulated interaction between RIP1 and Flag-Pellino1 without altering RIP1 and TRIF interaction, which is accompanied by a significant enhancement of RIP1 and caspase-8 interaction (Figure S2F). These results led us to the proposal that hypoxia might destabilize Pellino1, which in turn releases TRIF-RIP1 signalosome to recruit caspase-8 and ultimately facilitates the LPS-dependent tubular cell apoptosis.

**Figure 3:**
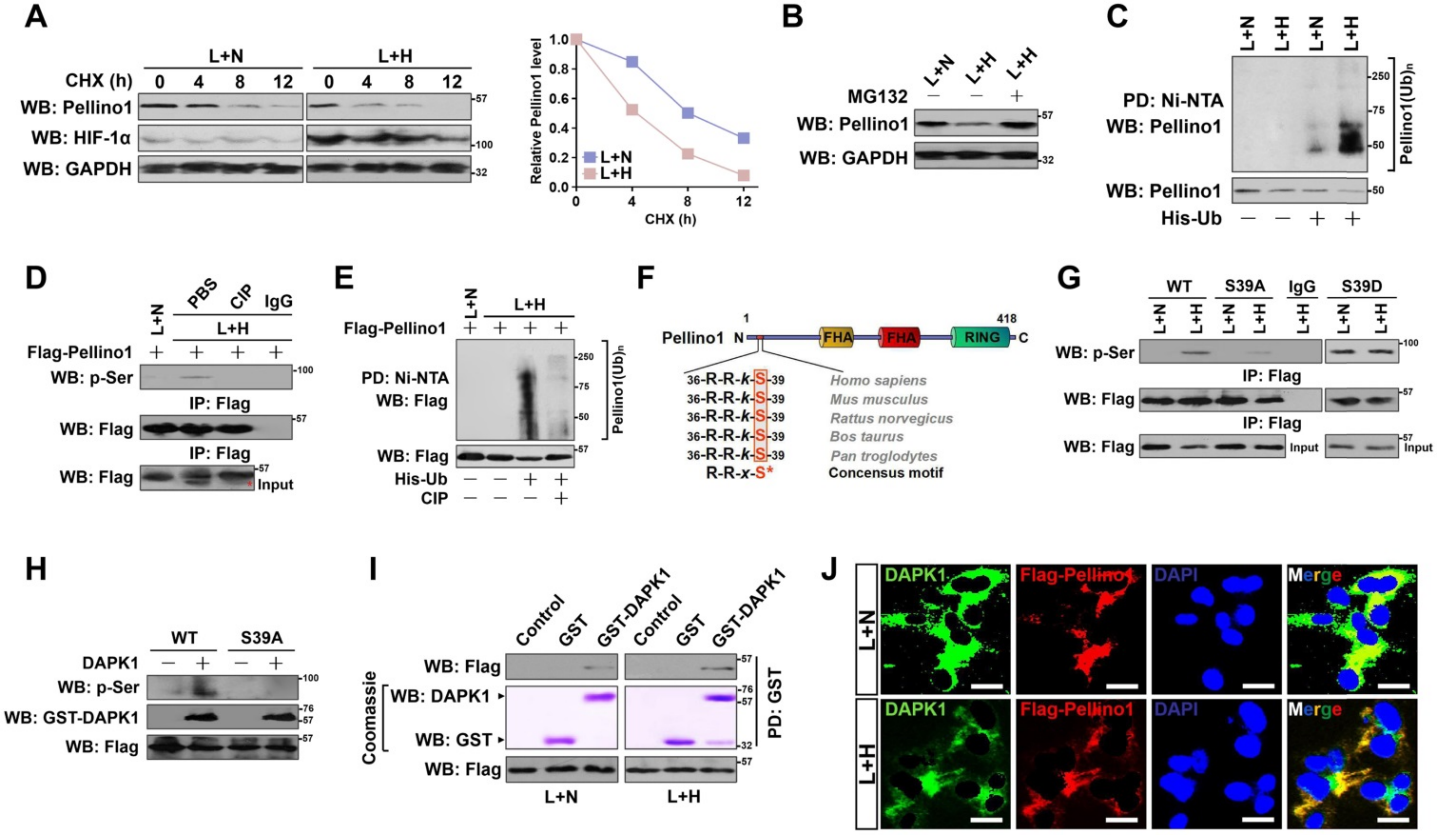
** DAPK1 directly interacts with and phosphorylates Pellino1 at Ser39 following hypoxia during LPS stimuli.** (A) CHX pulse-chase experiments determining the turnover of Pellino1 protein in LPS-stimulated HK-2 cells with or without hypoxia exposure in the presence or absence of 20 µg/mL CHX treatment for the indicated times. (B) Western blotting analyses testing abundance of Pellino1 protein in LPS-stimulated HK-2 cells with or without hypoxia exposure in the presence or absence of 10 µM MG132 treatment. (C) Cellular ubiquitination assays comparing the polyubiquitylation levels of Pellino1 in LPS-stimulated HK-2 cells with or without hypoxia exposure. (D) Coimmunoprecipitation assay assessing the abundance of Pellino1 Ser phosphorylation in Flag-tagged wild-type Pellino1-expressed HK-2 cells exposed to 50 ng/mL LPS with or without hypoxia in the presence of CIP treatment. (E) Cellular ubiquitination assays comparing the poly-Ub levels of Pellino1 in Flag-tagged wild-type Pellino1-expressed HK-2 cells exposed to 50 ng/mL LPS with or without hypoxia in the presence of CIP treatment. (F) Sequence alignment of DAPK1 consensus phosphorylation motif R-R-*x-*S* in Pellino1 among different species. (G) Coimmunoprecipitation assay determining the amount of Pellino1 Ser phosphorylation in Flag-tagged wild-type Pellino1 (WT)-, mutant Pellino1 Ser39A (S39A)- or Pellino1 Ser39D (S39D)-expressed HK-2 cells exposed to 50 ng/mL LPS with or without hypoxia. (H) *In vitro* protein kinase assay with mixing purified DAPK1 protein and IPs of Flag-tagged wild-type Pellino1 (WT) or mutant Pellino1 Ser39A (S39A) followed by WB analyses with an anti-pSer antibody. (I) GST-pulldown assay with mixing GST-DAPK1 kinase and IPs of Flag-tagged wild-type Pellino1 from HK-2 cells followed by WB analyses with an anti-DAPK1 antibody. PD: pull-down. (J) Representative immunfluorescence images detecting the localization of DAPK1 and Pellino1 in Flag-tagged wild-type Pellino1-expressed HK-2 cells exposed to 50 ng/mL LPS with or without hypoxia. Scale bar = 25 µm.

We next enrolled cycloheximide (CHX) pulse-chase experiments to test whether hypoxia destabilizes Pellino1 in the LPS-stimulated cells. To this end, we treated HK-2 cells with CHX and the turnover of endogenous Pellino1 (termed as ePellino1 hereafter) was monitored. The ePellino1 protein levels from LPS-stimulated cells were gradually decreased after CHX treatment in a time-dependent manner as expected; however, more striking turnover of ePellino1 was observed in the LPS-stimulated cells that were concomitantly incubated under hypoxia (Figure 3A). Our data reveal that ubiquitin (Ub)-proteasome system (UPS) might be the central mechanism for degrading Pellino1 protein because pretreatment of the LPS plus hypoxia-costimulated HK-2 cells with proteasome inhibitor MG132 completely restored ePellino1 abundance to the levels as seen in the LPS-stimulated cells (Figure 3B). In support of this, cellular ubiquitination assay of the His-tagged Ub protein immobilized on Ni^2+^-nitrilotriacetic acid (NTA)-sepharose beads with an anti-Pellino1 antibody depicted that hypoxia substantially augmented polyubiquitylation of Pellino1, as judged by the increased poly-Ub conjugation of ePellino1 in the LPS plus hypoxia-costimulated cells in contrast to the cells with LPS single stimuli (Figure 3C). These data imply that hypoxia destabilizes Pellino1 by UPS during LPS stimuli.

### DAPK1 directly interacts with and phosphorylates Pellino1 at Ser39 in response to hypoxia during LPS stimuli

Next, we sought to investigate the underlying mechanisms of how hypoxia triggers Pellino1 polyubiquitylation and degradation during LPS stimuli. Ubiquitylation and phosphorylation are two most common posttranslational modifications for intracellular proteins 34. The lysine residues of substrate proteins undergo ubiquitylation modification often following their serine residues are phosphorylated 35. Therefore, it is plausible that Pellino1 protein needs to be phosphorylated before being polyubiquitylated by hypoxia during LPS stimuli. Treatment of the LPS plus hypoxia-costimulated cells with an alkaline phosphatase (calf intestinal phosphatase [CIP]) robustly abolished the Ser phosphorylation of Pellino1 as determined by WB analyses of the immunoprecipitated Flag-Pellino1 protein using a phospho-Ser antibody (Figure 3D), which kinetically correlated with a lower poly-Ub conjugation of Pellino1 than that in the costimulated cells without CIP treatment (Figure 3E). CIP treatment successfully ameliorated Pellino1degradation caused by hypoxia in the LPS-stimulated cells, implicating that the hypoxia-dependent phosphorylation of Pellino1 protein might be critical for its polyubiquitylation and turnover upon LPS stimuli.

To identify the putative residue within the Pellino1 protein that is phosphorylated by hypoxia upon LPS stimuli, we analyzed the amino acid (aa) sequence of Pellino1 and found that N-terminus of Pellino1 contains an evolutionarily conserved peptide (^36^RRkS^39^) which matches the death associated protein kinase 1 (DAPK1) consensus phosphorylation motif R-R-*x-*S* (*x* represents any aa and *denotes the phosphorylated residue) (Figure 3F), raising the possibility that DAPK1 is the possible kinase responsible for phosphorylating Pellino1. To verify whether DAPK1 phosphorylates Pellino1, we treated the LPS plus hypoxia-costimulated cells with DAPK1 inhibitor (DAPK1-i). Administration of DAPK1-i largely perturbed Ser phosphorylation of Pellino1 (Figure S3A). Likewise, WB of immunoprecipitated Flag-Pellino1 with the anti-phospho-Ser antibody delineated that transfection with DAPK1 siRNA, which efficiently depleted DAPK1 as reflected by the downregulated protein levels, instead of control siRNA, fully blocked Pellino1 Ser phosphorylation in the LPS plus hypoxia-costimulated cells (Figure S3B-C). To directly address whether hypoxia induces phosphorylation of Pellino1 at Ser39 during LPS stimuli, we introduced the mutant Pellino1 S39A (in which serine 39 was mutated into a phospho-defective alanine [A]) into the LPS plus hypoxia co-stimulated cells. Figure 3G showed that the mutant Pellino1 S39A, rather than its wild-type counterpart, greatly mitigated the hypoxia-induced Ser phosphorylation upon LPS stimuli. In contrast, mutation of Ser39 into a phospho-mimetic aspartic acid (D) resulted in higher levels of Pellino1 phosphorylation in the LPS-stimulated cells irrespective of hypoxic status. The mutant Pellino1 S39A, but not wild-type Pellino1, lost the ability to undergo the DAPK1-induced Ser phosphorylation in an *in vitro* kinase assay (Figure 3H), indicating that DAPK1 phosphorylates Pellino1 at Ser39 during LPS stimuli under hypoxia.

Examination of the Flag-tagged wild-type Pellino1 protein immunoprecipitating from the LPS plus hypoxia-costimulated cells followed by WB analyses with an anti-DAPK1 antibody identified an apparent interaction between DAPK1 and Pellino1 (Figure S3D). An *in vitro* glutathione *S*-transferase (GST) pulldown assays with mixing purified GST-DAPK1 and IPs of Flag-tagged wild-type Pellino1 from the LPS plus hypoxia-costimulated cells validated direct interaction between the two proteins, which was evidenced by the observation that GST-DAPK1 but not GST was able to bind to Pellino1, and GST-DAPK1 more strongly binded to Pellino1 from cells with LPS plus hypoxia costimuli than from cells stimulated with LPS alone (Figure 3I). Immunofluorescent analyses showed that both DAPK1 and Pellino1 exhibited a diffuse, cytoplasmic staining pattern and colocalized with each other following LPS plus hypoxia costimuli (Figure 3J). Generally, our data suggest that hypoxia activates DAPK1 to interact with Pellino1 during LPS stimuli.

### DAPK1-mediated Pellino1 Ser39 phosphorylation contributes to Pellino1 turnover, which is instrumental for the LPS-induced caspase-8 recruitment of TRIF-RIP1 signalosome and tubular damage under hypoxia

**Figure 4:**
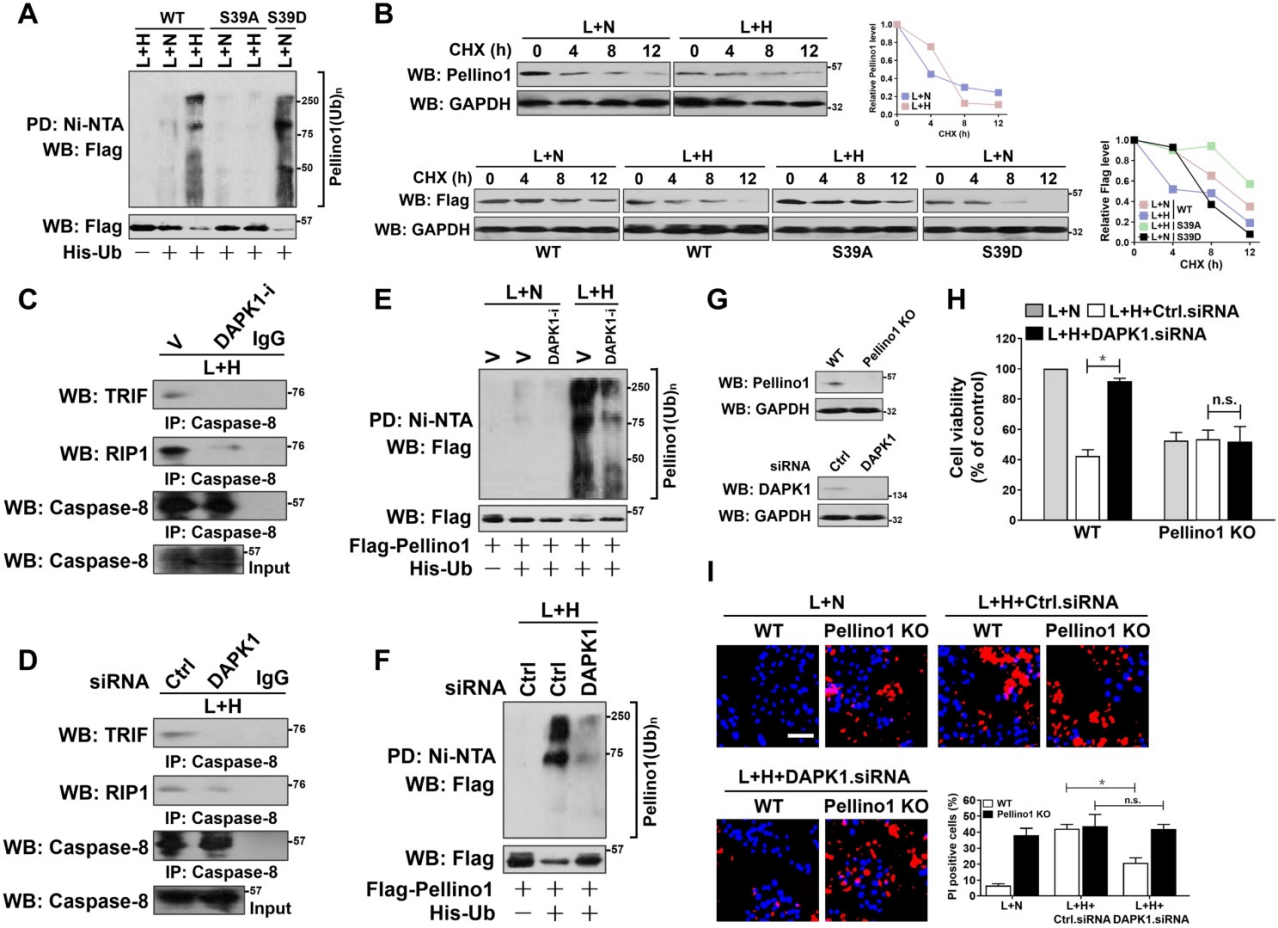
** DAPK1-mediated Pellino1 Ser39 phosphorylation contributes to Pellino1 turnover, which is instrumental for the LPS-induced caspase-8 recruitment of TRIF-RIP1 signalosome and tubular damage under hypoxia.** (A) Cellular ubiquitination assays comparing the poly-Ub levels of Pellino1 in Flag-tagged wild-type Pellino1 (WT)-, mutant Pellino1 Ser39A (S39A)- or Pellino1 Ser39D (S39D)-expressed HK-2 cells exposed to 50 ng/mL LPS with or without hypoxia. (B) CHX pulse-chase experiments determining the turnover of Pellino1 protein in Flag-tagged wild-type Pellino1 (WT)-, mutant Pellino1 Ser39A (S39A)- or Pellino1 Ser39D (S39D)-expressed HK-2 cells exposed to 50 ng/mL LPS with or without hypoxia in the presence or absence of 20 µg/mL CHX treatment for the indicated times. (C) Coimmunoprecipitation assay measuring the interaction of caspase-8 and TRIF and RIP1 in HK-2 cells exposed to 50 ng/mL LPS with or without hypoxia in the presence of 10 µM DAPK1 inhibitor (DAPK1-i) treatment. V, vehicle. (D) Coimmunoprecipitation assay examining the interaction of caspase-8 and TRIF and RIP1 in HK-2 cells exposed to 50 ng/mL LPS with or without hypoxia in the presence of DAPK1 siRNA transfection. (E) Cellular ubiquitination assays comparing the poly-Ub levels of Pellino1 in Flag-tagged wild-type Pellino1 (WT)-expressed HK-2 cells exposed to 50 ng/mL LPS with or without hypoxia in the presence of 10 µM DAPK1 inhibitor (DAPK1-i) treatment. (F) Cellular ubiquitination assays comparing the poly-Ub levels of Pellino1 in Flag-tagged wild-type Pellino1 (WT)-expressed HK-2 cells exposed to 50 ng/mL LPS with or without hypoxia in the presence of DAPK1 siRNA transfection. (G) *Top panel:* Western blotting analyses examining abundance of Pellino1 protein in Pellino1 knockout HK-2 cells (Pellino1 KO) where Pellino1 was deleted from the genome by CRISPR-Cas9 editing.* Bottom panel:* Western blotting analyses evaluating levels of DAPK1 protein expression in HK-2 cells with or without DAPK1 siRNA transfection. (H) MTT assay comparing cell viability of Pellino1 knockout HK-2 cells exposed to 50 ng/mL LPS with or without hypoxia in the presence or absence of DAPK1 siRNA transfection. Experiments were performed three times and data are expressed as mean ± s.d. *P<0.05, one-way ANOVA, post hoc comparisons, Tukey's test. n.s. no significant. (I) Representative pictures and quantification from Hoechst33342 and PI double-staining assay of Pellino1 knockout HK-2 cells exposed to 50 ng/mL LPS with or without hypoxia in the presence or absence of DAPK1 siRNA transfection. Experiments were performed three times and data are expressed as mean ± s.d. *P<0.05, one-way ANOVA, post hoc comparisons, Tukey's test.

To determine whether phosphorylation at Ser39 is necessary for Pellino1 turnover, HK-2 cells harboring Flag-tagged wild-type Pellino1, mutant Pellino1 S39A or S39D were costimulated with LPS and hypoxia. Figure 4A showed that the mutant Pellino1 S39A was resistant to the hypoxia-induced polyubiquitylation and degradation when compared with the wild-type Pellino1 upon LPS stimuli, whereas the mutant Pellino1 S39D, which mimics well the DAPK1-phosphorylated status under hypoxia, had higher polyubiquitylation levels and lower protein abundance even in the case of LPS single stimuli. In the CHX pulse-chase analysis, Pellino1 protein kept stable during the course of LPS plus hypoxia costimuli when Ser39 was mutated into the non-phosphorylatable alanine. By contrary, mutation of Ser39 into the phospho-mimetic aspartic acid appreciably accelerated Pellino1 protein turnover in the presence of LPS single stimuli (Figure 4B). These data implicate that Ser39 phosphorylation of Pellino1 induced by hypoxia destabilizes Pellino1 upon LPS stimuli.

Along with an attenuation of caspase-8 assembly with TRIF and RIP1 (Figure 4C), deactivation of DAPK1 using DAPK1-i in the LPS plus hypoxia-costimulated cells restored the interaction of TRIF and RIP1 with Pellino1 (Figure S3A), suggesting that DAPK1 plays a principal role in the hypoxia-induced caspase-8 recruitment of TRIF-RIP1 signalosome during LPS stimuli, for which the Ser39 phosphorylation-mediated Pellino1 polyubiquitylation and turnover might be essential. The principal role of DAPK1 in the hypoxia-dependent caspase-8 recruitment of TRIF-RIP1 signalosome upon LPS stimuli was further confirmed by the DAPK1 siRNA-transfected cells, which displayed decreased TRIF and RIP1 abundance in IPs of caspase-8 in contrast to the cells transfected with control siRNA (Figure 4D). Meanwhile, depletion of DAPK1 with siRNA abrogated the LPS plus hypoxia-induced disassembly of Pellino1 from TRIF and RIP1 (Figure S3C). Deactivating, silencing or knocking out DAPK1 impaired the ability of hypoxia to trigger Pellino1 polyubiquitylation and turnover during LPS stimuli (Figure 4E-F, Figure S3E-F), presumably resulting from the reduced Ser39 phosphorylation. Thus, DAPK1 phosphorylates Pellino1 at Ser39, evoking its polyubiquitylation and turnover and thus releasing TRIF-RIP1 signalosome to recruit caspase-8.

To pursue whether Pellino1 turnover is the critical mechanism underlying the pro-apoptotic phenotypes of DAPK1, we transfected the Pellino1-knockout HK-2 cells with DAPK1.siRNA and stimulated them with LPS plus hypoxia. As depicted in Figure 4G-I, depletion of DAPK1 successfully alleviated apoptosis of HK-2 cells elicited by LPS plus hypoxia (*P <* 0.05), but this did not occur when Pellino1 had been knocked out (n.s.), underscoring a key role of Pellino1 turnover in the apoptosis-inducing phenotypes of DAPK1 in tubular cells during LPS stimuli under hypoxic conditions.

### DAPK1 is a potential therapeutic target for tubular damage in septic AKI

**Figure 5:**
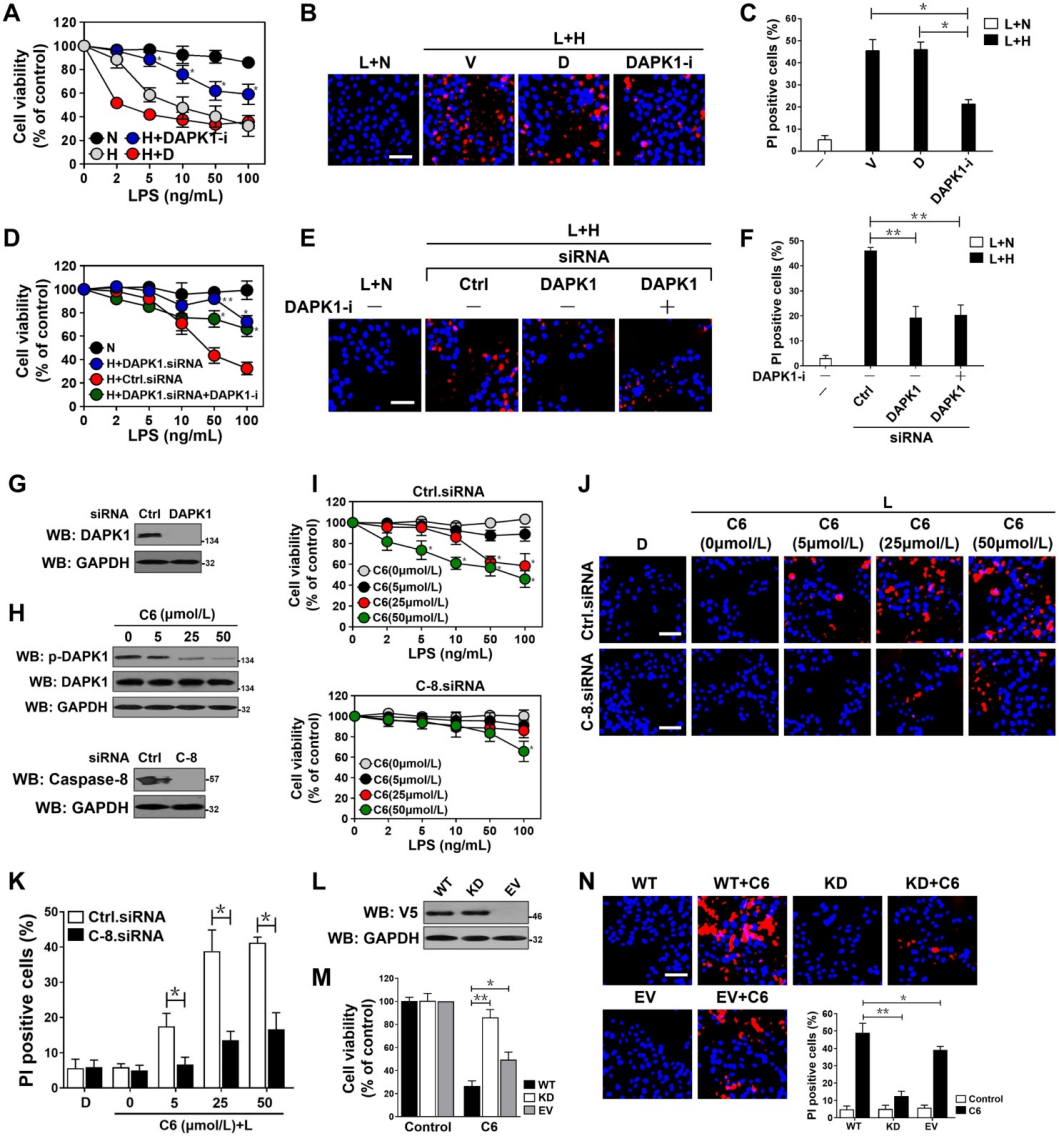
** DAPK1 plays a principal role in the LPS-induced tubular damage under hypoxia.** (A) MTT assay measuring cell viability of HK-2 cells exposed to LPS with or without hypoxia in the presence of 10 µM DAPK1 inhibitor (DAPK1-i) treatment. Experiments were performed three times and data are expressed as mean ± s.d. Experiments were performed three times and data are expressed as mean ± s.d. *P<0.05 versus H+D, one-way ANOVA, post hoc comparisons, Tukey's test. (B and C) Representative pictures (B) and quantification (C) from Hoechst33342 and PI double-staining assay of HK-2 cells exposed to 50 ng/mL LPS with or without hypoxia in the presence of 10 μM DAPK1 inhibitor (DAPK1-i) treatment. Experiments were performed three times and data are expressed as mean ± s.d. *P<0.05 one-way ANOVA, post hoc comparisons, Tukey's test. (D) MTT assay assessing cell viability of HK-2 cells exposed to LPS with or without hypoxia in the presence of DAPK1 siRNA transfection plus 10 µM DAPK1 inhibitor (DAPK1-i) treatment. Experiments were performed three times and data are expressed as mean ± s.d. *P<0.05 and **P<0.01 versus H+Ctrl.siRNA, one-way ANOVA, post hoc comparisons, Tukey's test. (E and F) Representative pictures (E) and quantification (F) from Hoechst33342 and PI double-staining assay of HK-2 cells exposed to 50 ng/mL LPS with or without hypoxia in the presence of DAPK1 siRNA transfection plus 10 µM DAPK1 inhibitor (DAPK1-i) treatment. Experiments were performed three times and data are expressed as mean ± s.d. **P<0.01, one-way ANOVA, post hoc comparisons, Tukey's test. (G) Western blotting analyses testing levels of DAPK1 protein expression in HK-2 cells with or without DAPK1 siRNA transfection. (H) *Top panel:* Western blotting analyses determining amount of DAPK1 Ser308 phosphorylation in HK-2 cells with or without the indicated concentrations of C6-ceramide (C6) treatment. *Bottom panel:* Western blotting analyses comparing levels of caspase-8 protein expression in HK-2 cells with or without caspase-8 siRNA transfection. (I) MTT assay measuring cell viability of HK-2 cells exposed to LPS with or without C6-ceramide (C6) treatment ranging from 0 to 50 µM in the presence of caspase-8 siRNA transfection. Experiments were performed three times and data are expressed as mean ± s.d. *P<0.05 versus 0 µM, one-way ANOVA, post hoc comparisons, Tukey's test. (J and K) Representative pictures (J) and quantification (K) from Hoechst33342 and PI double-staining assay of HK-2 cells exposed to 50 ng/mL LPS with or without 50 µM C6-ceramide (C6) treatment in the presence of caspase-8 siRNA transfection. Experiments were performed three times and data are expressed as mean ± s.d. *P<0.05, one-way ANOVA, post hoc comparisons, Tukey's test. (L) Western blotting analyses examining levels of V5-DAPK1 protein in HK-2 cells with V5-tagged wild-type or kinase-dead (KD) DAPK1 transfection. EV, empty vector. (M) MTT assay evaluating cell viability of HK-2 cells with or without 50 µM C6-ceramide (C6) treatment in the presence of V5-tagged wild-type or kinase-dead (KD) DAPK1 transfection. Experiments were performed three times and data are expressed as mean ± s.d. *P<0.05 and **P<0.01, one-way ANOVA, post hoc comparisons, Tukey's test. (N) Representative pictures and quantification from Hoechst33342 and PI double-staining assay of HK-2 cells with or without 50 µM C6-ceramide (C6) treatment in the presence of V5-tagged wild-type or kinase-dead (KD) DAPK1 transfection. Experiments were performed three times and data are expressed as mean ± s.d. *P<0.05 and **P<0.01, one-way ANOVA, post hoc comparisons, Tukey's test.

Take into account that DAPK1 releases TRIF-RIP1 signalosome to recruit caspase-8 via inducing Pellino1 phosphorylation and polyubiquitylation, we wondered whether DAPK1 participates in the pro-apoptotic phenotypes of LPS under hypoxia. To address this issue, we examined the proportion of tubular dying in the LPS plus hypoxia-costimulated HK-2 cells in the presence or absence of DAPK1-i treatment. As shown in Figure 5A-C, LPS plus hypoxia enhanced the magnitude of tubular apoptosis that could be reversed by DAPK1-i pretreatment (*P <*0.05). Although LPS plus hypoxia induced apoptosis in HK-2 cells transfected with control siRNA, it was unable to do so in cells that were transfected with DAPK1 siRNA (*P <*0.05 and *P <*0.01, Figure 5D-G). Inhibition of DAPK1 did not further enhance the ability of DAPK1 siRNA to prevent the LPS plus hypoxia-costimulated cell apoptosis, which may attribute to the fact that little or no druggable target was present in these cells. To exclude the possibility of off-target impact of DAPK1 deficiency on suppressing the LPS-dependent tubular apoptosis under hypoxia, we asked whether the anti-apoptotic phenotypes of DAPK1 deficiency could be rescued by re-expression of DAPK1. For this purpose, we transfected HK-2 cells with DAPK1.shRNA (sh.DAPK1), followed by reconstituting the DAPK1-deficient cells with a V5-tagged wild-type DAPK1 and then stimulated them with LPS plus hypoxia. Exogenous downregulation of DAPK1 counteracted the LPS plus hypoxia-induced apoptosis of HK-2 cells, but this effect could be overriden by concurrent expression of DAPK1 (*P <*0.05, Figure S4A-D). These findings suggest that DAPK1 might qualify as a potential therapeutic target for the LPS-dependent tubular damage under hypoxic conditions.

To explore the role of DAPK1 activity in the LPS-induced tubular apoptosis, we treated the LPS-stimulated HK-2 cells with the DAPK1 agonist C6-ceramide (C6) 36. DAPK1 activation, as indicated by dephosphorylation at Ser308, dose-dependently occurred after treatment with C6 (Figure 5H). Activation of DAPK1 by C6 apparently elevated apoptosis of the LPS-stimulated HK-2 cells in a caspase-8-dependent manner as seen under hypoxia (*P <*0.05, Figure 5I-K). C6 treatment exaggerated apoptotic phenotypes in the LPS-stimulated HK-2 cells expressing wild-type DAPK1, which had little effect on cell death in the presence of LPS single stimuli. In contrast, overexpressing a kinase dead (KD) DAPK1 mutant in the LPS-stimulated HK-2 cells impaired the ability of C6 to induce apoptosis (*P <*0.05 and *P <*0.01, Figure 5L-N). In agreement with the pro-apoptotic roles of DAPK1 kinase activity during LPS stimuli, the LPS plus hypoxia-induced apoptosis of HK-2 cells was correlated with a drop in DAPK1 Ser308 phosphorylation that could be recovered after withdrawal of hypoxic exposure (Figure S4E). However, the cells with LPS plus hypoxia costimuli displayed no significant changes in death rate following either transient or prolonged withdrawal of hypoxia (Figure S4F-G), excluding irreversible effects. These data suggest that DAPK1 activation is crucial for the irreversible tubular apoptosis caused by LPS and hypoxia costimuli.

**Figure 6:**
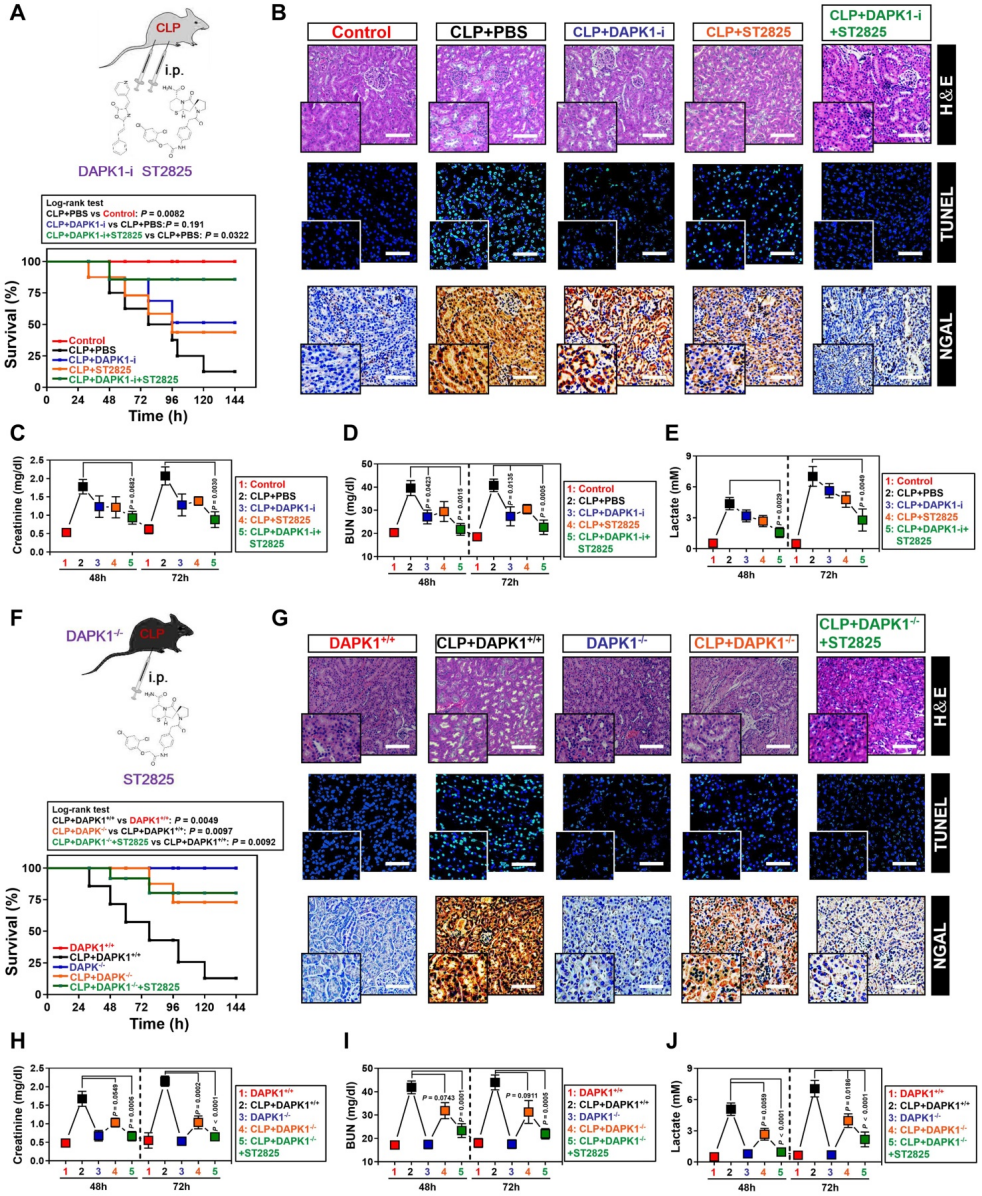
** Pharmacological deactivation or genetic ablation of DAPK1 synergizes with MyD88 inhibitor to protect mice against septic AKI.** (A) Kaplan-Meier method analyzing survivals of CLP-treated mice with intraperitoneal (i.p.) administration of PBS, 1 mg/kg DAPK1-i and 0.2 mg ST2825 or both at the indicated times after intraabdominal sepsis induction (*n* = 8 per group). Log-rank t test was used to caculate the P value. (B) Representative H&E, TUNEL and NGAL staining images in kidney tissues from CLP-treated mice with intraperitoneal (i.p.) administration of PBS, 1 mg/kg DAPK1-i and 0.2 mg ST2825 or both at 72 h after intraabdominal sepsis induction. (C-E) Serum creatinine (Scr, C), blood urea nitrogen (BUN, D) and lactate (E) levels from CLP-treated mice with intraperitoneal (i.p.) administration of PBS, 1 mg/kg DAPK1-i and 0.2 mg ST2825 or both at the indicated times after intraabdominal sepsis induction (*n* = 8 per group). One-way ANOVA post hoc comparisons with tukey's test was used to caculate the P value. (F) Kaplan-Meier method analyzing survivals of CLP-treated DAPK1^-/-^ mice with or without intraperitoneal (i.p.) administration of 0.2 mg ST2825 at the indicated times after intraabdominal sepsis induction (*n* = 10 per group). Log-rank t test was used to caculate the P value. (G) Representative H&E, TUNEL and NGAL staining images in kidney tissues from CLP-treated DAPK1^-/-^ mice with or without intraperitoneal (i.p.) administration of 0.2 mg ST2825 at 72 h after intraabdominal sepsis induction. (H-J) Serum creatinine (Scr, H), blood urea nitrogen (BUN, I) and lactate (J) levels from CLP-treated DAPK1^-/-^ mice with or without intraperitoneal (i.p.) administration of 0.2 mg ST2825 at the indicated times after intraabdominal sepsis induction (*n* = 10 per group). One-way ANOVA post hoc comparisons with tukey's test was used to caculate the P value.

The ability of DAPK1 inhibition or depletion to prevent the LPS-induced tubular apoptosis under hypoxia *in vitro* prompted us to dissect whether inhibition or depletion of DAPK1 counteracts septic AKI, the thorny clinical disease characterized by systemic inflammatory response syndrome (SIRS) in conjunction with ischemic/hypoxic tubular damage. To test this possibility, we established septic mice models using cecal ligation and puncture (CLP, Figure 6A, top panel) and intraperitoneally administered them with DAPK1-i in 24+, 48+, 72+ hours following onset of CLP, respectively. Kaplan-Meier survival revealed that either DAPK1-i delayed the septic lethality since the survival ratios in the CLP-treated mice received DAPK1-i injection were higher than those in the treated mice received PBS injection (P = 0.191, Log-rank test, Figure 6A, bottom panel). As observed in hematoxylin and eosin (H&E) staining, the CLP-treated animals developed kidney disease with characteristic features of acute kidney injury (AKI), including loss of epithelial brush border, tubular epithelial vacuolization and epithelial desquamation, while DAPK1-i administration efficiently alleviated these phenotypes (Figure 6B). Histopathological analysis of kidney tissues from mice at 72h after CLP showed the DAPK1-i-injected mice presented a significantly reduced number of cells positive for TdT-mediated dUTP nick end labelling (TUNEL) staining, an indicative of apoptotic response, and cells positive for neutrophil gelatinase-associated lipocalin (NGAL), an index of renal injury, compared to the PBS-injected or si.Ctrl-delivered mice (Figure 6B), respectively, suggesting that deactivation of DAPK1 allows mice resistant to the sepsis-initiated AKI. Additional evidence that DAPK1 deactivation protects mice against septic AKI was obtained from the biochemical detection, where the levels of serum creatinine (Scr), blood urea nitrogen (BUN) and lactate from CLP-treated animals with DAPK1-i injection were much less than those from their littermates with PBS injection (Figure 6C-E).

As a complementary approach to these DAPK1 deactivation studies, we asked whether genetic ablation of DAPK1 can prevent septic AKI. To this end, DAPK1 knockout (DAPK1^-/-^) mice were subjected to CLP (Figure 6F, top panel). Compared with their littermates, DAPK1^-/-^ mice with CLP had enhanced median survival duration (P = 0.0097, Log-rank test, Figure 6F, bottom panel), reduced tubular damage and increased clearance rate of Scr, BUN and lactate (Figure 6G-J).

### Pharmacological deactivation or genetic ablation of DAPK1 synergizes with MyD88 inhibitor to protect mice against septic AKI

ST2825 is a selective MyD88 homodimerization inhibitor that attenuates pro-inflammatory cytokine production in human monocytic cells and protects mice against ischemia/reperfusion (I/R) injuries 37-39. Considering the potential therapeutic capacity based on DAPK1 deactivation or ablation in CLP mice, we hypothesized that management of septic AKI might further benefit from the combination therapy with DAPK1-i and ST2825. Indeed, although either DAPK1-i or ST2825 treatment alone had little effect on IL-6 secretion (Figure S5A) and percentage of PI-positive apoptotic staining (Figure S5B-C) of the LPS plus hypoxia-costimulated cells, respectively, cotreatment of ST2825 and DAPK1-i led to a significant decline in both of these two phenotypes (*P <*0.05, Figure S5). Administration of mice with DAPK1-i plus ST2825 conferred more prominent protection against lethality than single treatment (P = 0.0322, Log-rank test, Figure 6A), quantitatively corresponding to the lowest tubulotoxicity, TUNEL-positive staining and NGAL levels in kidney tissues (Figure 6B). Consistent with the data described for AKI initiated by sepsis, examination of the CLP-treated mice with DAPK1-i plus ST2825 administration identified an additive decline in Scr, BUN and lactate levels (Figure 6C-E). DAPK1-i treatment with ST2825 or both did not result in any overt signs of toxicity as reflected by weight loss in all mice models tested. As expected, ST2825 further prolonged survival, alleviated kidney injury and reduced Scr, BUN and lactate levels in the DAPK1^-/-^ mice with CLP (Figure 6F-J). These findings highlighted that deactivation or ablation of DAPK1 synergizes with MyD88 inhibitor to prevent septic AKI in mice.

## Discussion

It becomes increasingly clear that severe pro-/anti-inflammatory disorders couple to ischemic/hypoxic tubular damage in the development and progression of septic AKI, the life-threatening clinical disease characterized by systemic inflammatory response syndrome (SIRS), microcirculation dysfunction and kidney failure 40, 41. Despite considerable advancements have been made in the past decade, the genetic and molecular nature of interdependence between inflammation and ischemic/hypoxic tubular damage during septic AKI remains elusive. As the prototypical component of endotoxin, LPS is known to play an important role in manipulating inflammatory responses, which has been intensively studied ever since it was discovered. Although emerging evidences indisputably show that LPS evokes extrinsic/intrinsic cell death pathways in a context-dependent manner, its apoptosis-inducible feature with respect to hypoxia is still not comprehensively studied. We provide demonstration here that the Ser39 phosphorylation-primed polyubiquitination and turnover of Pellino1 by DAPK1 is a prerequisite for TRIF-RIP1 signalosome to gain the ability to recruit caspase-8, which in turn contributes to the LPS-dependent tubular apoptosis under hypoxic circumstances. Our future work will aim at ascertaining whether other redox-responsive signaling molecules are essential for the inflammation-coupling tubular damage upon LPS plus hypoxia costimuli and exploring the precise mechanism of how endotoxin exasperates AKI under hypoxic microenvironment.

Dephosphorylation of DAPK1 at Ser308 reflects the catalytic activity of DAPK1; ischemic injury activates DAPK1 and dephosphorylates DAPK1 21, 42. Our data show that DAPK1 Ser308 phosphorylation is impaired when DAPK1 is activated by C6-ceramide. We also observe that phosphorylation of DAPK1 at Ser308 in the LPS plus hypoxia-costimulated tubular cells can be recovered after hypoxia withdrawal. Our previous study showed that RIP1 deubiquitination links hypoxic status to apoptosis of renal cells 27. Pellino1 is known to attach the poly-ubiquitin chains into RIP1, this may account for the increased apoptosis ratio in the Pellino1-deficient tubular cells during LPS stimuli. In line with this finding, biochemical results validate that depleting Pellino1 in the LPS-stimulated tubular cells facilitates caspase-8 recruitment by TRIF and RIP1. Herein, the most parsimonious explanation for our observation is that under inflammatory circumstances, two distinct LPS-responsive pathways are generated: the first one is that NF-κB activation as a result of MyD88 signaling transduction elicits inflammatory response via producing IL-6; the second one is that Pellino1 interacts with TRIF-RIP1 signalosome and simultaneously ubiquitinates RIP1through its E3 ligase activity to maintain cell survival. Following hypoxia exposure, however, activated DAPK1 phosphorylates Pellino1 at Ser39, which triggers polyubiquitination and turnover of Pellino1, leading to TRIF-RIP1 signalosome release for caspase-8 recruitment and thereby coupling the LPS/MyD88-dependent inflammatory response to tubular damage (Figure S6). Additional distinction of the two LPS-responsive pathways is observed in renal endothelial cells (ECs), which contribute to myofibroblast generation through endothelial-to-mesenchymal transition (EndMT) and result in tubular damage during septic AKI. Coculture with adult renal stem/progenitor cells (ARPCs) abrogates the LPS-induced EndMT process in a MyD88-independent manner. This effect could be due to the enhanced secretion of C-X-C motif chemokine 6 (CXCL6), serum amyloid A-4 protein (SAA4) and BPI fold-containing family A member 2 (BPIFA2) by ARPCs as has been proposed previously 43.

Previous publications identify numerous substrates that can be phosphorylated by DAPK1 44, 45. To our knowledge, we believe our present study is the first to report that the DAPK1-inducible Pellino1 Ser39 phosphorylation is the central mechanism for polyubiquitylation and turnover of Pellino1. Mutation of Ser39 residue on Pellino1 into alanine, blocks its serine phosphorylation while does not influence its interaction with DAPK1 upon LPS single stimuli. Beside phosphorylation, other post-translational modifications (PTMs) of Pellino1 might be also critical for its function. In this regard, future work are warranted to test whether additional PTMs (methylation, acetylation and glycosylation, etc.), parallel to phosphorylation, are involved in regulating Pellino1 function and controlling the inflammation-coupling damage when tubular cells lie in an inflammatory/hypoxic state.

Administration of DAPK1 inhibitor or genetic knockout of DAPK1 prolongs survival, reverses exacerbation of AKI and accelerates Scr, BUN as well as lactate clearance in septic mice, implicating that DAPK1 is a promising therapeutic target to manage septic AKI. Because DAPK1 inhibitor also blocks the hypoxia-dependent Pellino1 Ser39 phosphorylation, caspase-8 recruitment of TRIF-RIP1 signalosome and tubular apoptosis during LPS stimuli, it is possible that pharmacological deactivation or genetic ablation of DAPK1 protects mice from septic AKI via controlling these events. Of note, combined treatment of DAPK1 inhibitor with ST2825 or administration of ST2825 into DAPK1 knockout mice provides synergistic protection against septic AKI, supporting further investigation and development of innovative combination-targeting therapeutic strategy for improving the clinical outcome of patients with septic AKI.

There are some limitations in our work. First, although we clearly demonstrate that Pellino1 Ser39 phosphorylation directs the LPS-inducible tubular apoptosis in response to hypoxia, the possibility that multiple phosphorylation sites of Pellino1 engage in this process could not be ruled out. Explicit illustration of the regulatory mechanism underlying PTMs of Pellino1 will be helpful for uncovering the molecular phenotypes of tubular damage during septic AKI. Second, in addition to DAPK1, several signaling molecules, such as aquaporin-3 (AQP-3), transient receptor potential melastatin-7 (TRPM-7) and nod-like receptor protein (NLRP), have also been shown to worsen ischemic/hypoxic damage 46-48. Therefore it would be of interest to test whether these components participate in the inflammation-coupling tubular damage under hypoxia as mentioned in the current study.

In summary, our study gain insight into the mechanism focusing on the inflammatory determinant of tubular damage in conjunction with hypoxic stress, which may lay framework for intensive understanding of LPS's function not only in septic AKI but also in other critical illnesses. Last but not least, novel therapeutic strategies targeting DAPK1 and MyD88 for the treatment of patients with septic AKI are in order.

## Supplementary Material

Supplementary figures and tables.

## Abbreviations

SIRS: systemic inflammatory response syndrome
AKI: acute kidney injury
PAMPs: pathogen-associated molecular patterns
LPS: lipopolysaccharide
TLR4: toll-like receptor 4
MyD88: myeloid differentiation primary response gene 88
IRAK1: interleukin-1 receptor-associated kinase 1
TRAF6: TNF receptor-associated factor 6
TRIF: TIR domain-containing adapter-inducing interferon β
RIP1: receptor-interacting protein 1
DAPK1: death-associated protein kinase 1
RIPA: radio-immunoprecipitation assay
CLP: cecal ligation and puncture
TUNEL: TdT-mediated dUTP nick end labelling
zVAD-FMK: benzyloxycarbonyl- Val-Ala-Asp-fluoromethylketone
CIP: calf intestinal phosphatase
NGAL: neutrophil gelatinase-associated lipocalin
Scr: serum creatinine
BUN: blood urea nitrogen
PTMs: post-translational modifications
