# Supplementary material for: Redox DAPK1 destabilizes Pellino1 to govern inflammation-coupling tubular damage during septic AKI
Source: Theranostics. 2020 Sep 15;10(25):11479–96. doi: 10.7150/thno.49870 (PMC7546007; doi:10.7150/thno.49870)
Supplement: Supplementary file 1 — Supplementary figures and tables. [file thnov10p11479s1.pdf]

Redox DAPK1 destabilizes Pellino1 to govern inflammation-coupling tubular damage during septic AKI

Bang-Chuan Hu[1], Guo-Hua Wu[2], Zi-Qiang Shao[1], Yang Zheng[1], Jin-Quan Liu[1], Run Zhang[1], Jun Hong[1], Xiang-Hong Yang[1], Ren-Hua Sun[1], Shi-Jing Mo[1]*

[1]*Department of Intensive Care Unit, Zhejiang Provincial People's Hospital, People's Hospital of Hangzhou Medical College, Hangzhou 310014, Zhejiang, P.R.China*

[2]*Zhejiang University School of Medicine, Zhejiang University, Hangzhou 310029, Zhejiang, P.R.China*

Corresponding author:

*Shi-Jing Mo, MD, PhD

Department of Intensive Care Unit

Zhejiang Provincial People's Hospital

People's Hospital of Hangzhou Medical College

Hangzhou, Zhejiang 310014

P.R. China

Email: moshijing@hmc.edu.cn

Supplemental Materials

Page 2-8: Supplemental Figure legends

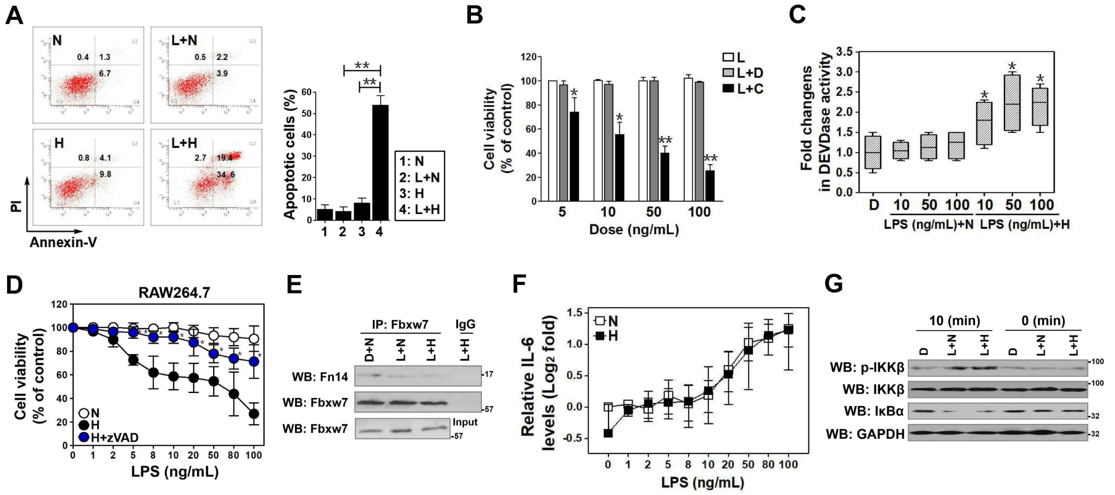

Supplemental Figure S1. Effects of LPS on tubular damage, DEVDase activities, NF-κB/IL-6 pathway and Fn14-SCF$^{Fbxw7\alpha}$ complex under hypoxia. (A) Representative histograms (left panel) and quantification (right panel) of flow cytometry with Annexin-V/PI staining in HK-2 cells exposed to 50 ng/mL LPS with or without hypoxia for 24 h. Experiments were performed three times and data are expressed as mean ± s.d. **, $P < 0.01$, one-way ANOVA, post hoc comparisons, Tukey's test. (B) MTT assay assessing cell viabilities of HK-2 cells exposed to LPS at the indicated concentrations with or without 0.6 mM $CoCl_2$ treatment. Experiments were performed three times and data are expressed as mean ± s.d. L: LPS, D: DMSO, C: $CoCl_2$. *, $P < 0.05$ and **, $P < 0.01$ versus L or L+D, one-way ANOVA, post hoc comparisons, Tukey's test. (C) DEVDase activity assays of HK-2 cells exposed to LPS at the indicated concentrations with or without hypoxia. Experiments were performed three times and data are expressed as mean ± s.d. *, $P < 0.05$ versus LPS+N, one-way ANOVA, post hoc comparisons, Tukey's test. (D) MTT assay comparing cell viability of RAW264.7 cells exposed to LPS at the indicated concentrations with or without hypoxia in the presence of 20 μM zVAD-FMK treatment. Experiments were performed three times and data are expressed as mean ± s.d. *, $P < 0.05$ versus H, one-way ANOVA, post hoc comparisons, Tukey's test. (E) Coimmunoprecipitation assay determining the interaction between Fn14 and Fbxw7α in LPS-stimulated HK-2 cells with or without hypoxia. (F) RT-qPCR analyses of IL-6 gene expression in HK-2 cells exposed to LPS at the indicated concentrations with or without hypoxia. Experiments were performed five times, each with quantitative

RT-PCR in technical duplicate and real-time values were normalized to glyceraldehyde 3-phosphate dehydrogenase (GAPDH). Data are expressed as mean ± s.d. (G) Western blotting analyses comparing levels of p-IKKβ and IκBα protein expression in LPS-stimulated HK-2 cells with or without hypoxia at the indicated times. GAPDH was used as internal control of cytoplasmic extractions.

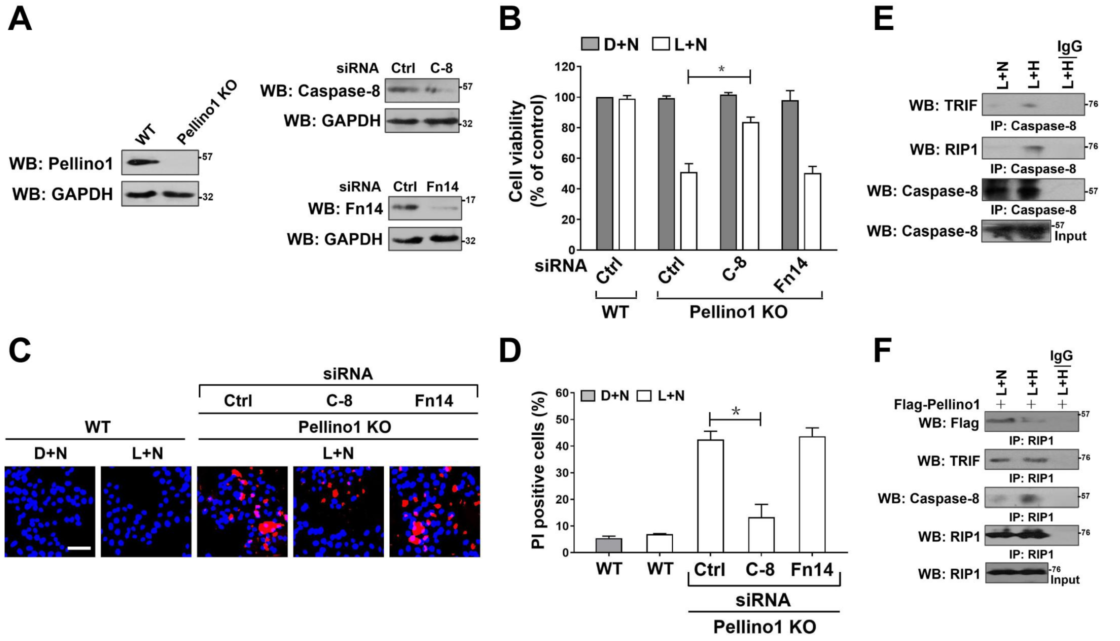

Supplemental Figure S2. Pellino1 deficiency mimicking hypoxia to induce caspase-8-mediated tubular damage upon LPS stimuli. (A) Western blotting analyses determining levels of Pellino1, caspase-8 or Fn14 protein expression in Pellino1 knockout HK-2 cells transfected with siRNA targeting caspase-8 or Fn14. (B) MTT assay measuring cell viability of Pellino1 knockout HK-2 cells exposed to 50 ng/mL LPS in the presence of siRNA targeting caspase-8 or Fn14 transfection. Experiments were performed three times and data are expressed as mean ± s.d. *, $P < 0.05$, one-way ANOVA, post hoc comparisons, Tukey's test. (C and D) Representative pictures (C) and quantification (D) from Hoechst33342 and PI double-staining assay of Pellino1 knockout HK-2 cells exposed to 50 ng/mL LPS in the presence of siRNA targeting caspase-8 or Fn14 transfection. Experiments were performed three times and data are expressed as mean ± s.d. *, $P < 0.05$, one-way ANOVA, post hoc comparisons, Tukey's test. (E) Coimmunoprecipitation assay comparing the interaction of caspase-8 with TRIF and RIP1 in LPS-stimulated HK-2 cells in the presence or absence of hypoxia exposure. (F) Coimmunoprecipitation assay determining the interaction of RIP1 with Flag-tagged wild-type Pellino1, TRIF and caspase-8 in LPS-stimulated HK-2 cells in the presence or absence of hypoxia exposure.

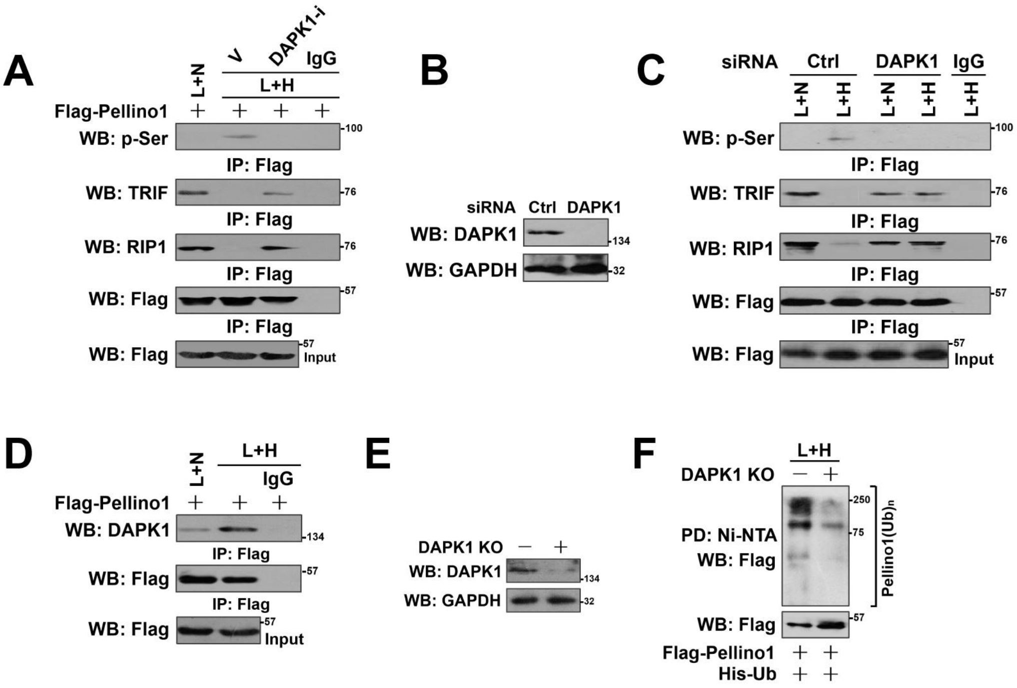

Supplemental Figure S3. Specificity of DAPK1 in mediating hypoxia-induced Pellino1 phosphorylation and disassociation from TRIF-RIP1 signalosome. (A) Coimmunoprecipitation assay examining the levels of Pellino1 Ser phosphorylation and the interaction of Pellino1 with TRIF and RIP1 in Flag-tagged wild-type Pellino1-expressed HK-2 cells exposed to 50 ng/mL LPS with or without hypoxia in the presence of 10 μM DAPK1 inhibitor (DAPK1-i) treatment. (B) Western blotting analyses detecting the abundance of DAPK1 in HK-2 cells with DAPK1 siRNA transfection. (C) Coimmunoprecipitation assay comparing the levels of Pellino1 Ser phosphorylation and the interaction of Pellino1 with TRIF and RIP1 in Flag-tagged wild-type Pellino1-expressed HK-2 cells exposed to 50 ng/mL LPS with or without hypoxia in the presence of DAPK1 siRNA transfection. (D) Coimmunoprecipitation assay measuring the interaction between DAPK1 and Pellino1 in Flag-tagged wild-type Pellino1-expressed HK-2 cells exposed to 50 ng/mL LPS with or without hypoxia. (E) Western blotting analyses examining levels of DAPK1 protein in DAPK1 knockout HK-2 cells (DAPK1 KO) where DAPK1 was deleted from the genome by CRISPR-Cas9 editing. (F) Cellular ubiquitination assays comparing the poly-Ub levels of Pellino1 in Flag-tagged wild-type Pellino1-expressed HK-2 cells exposed to 50 ng/mL LPS plus hypoxia with or without DAPK1 knockout.

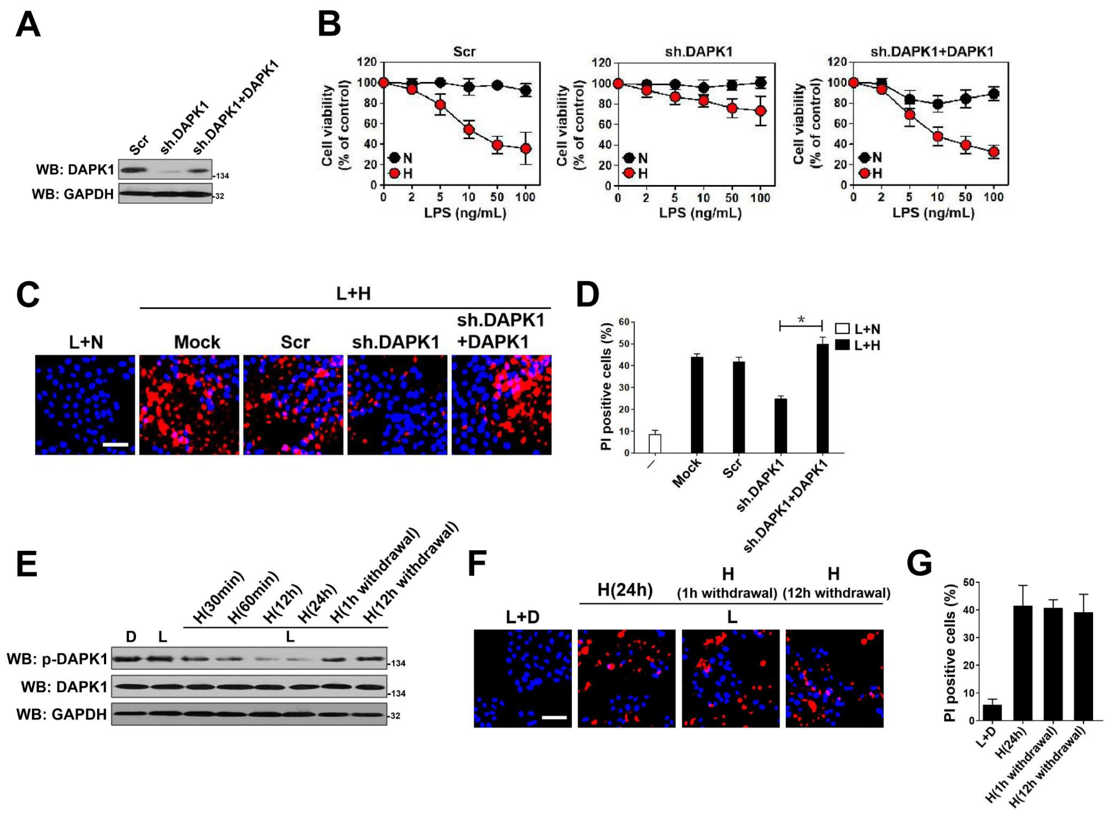

Supplemental Figure S4. The anti-apoptotic phenotype of DAPK1 deficiency could be reversed by re-expression of DAPK1. (A) Western blotting analyses assessing the abundance of DAPK1 in HK-2 cells with DAPK1 shRNA transfection in the presence of V5-tagged DAPK1 re-expression. Scr: scrambled shRNA. (B) MTT assay comparing cell viability of LPS-stimulated HK-2 cells with or without hypoxia in the presence of DAPK1 shRNA (sh.DAPK1) transfection or DAPK1 shRNA plus V5-tagged DAPK1 cotransfection. Experiments were performed three times and data are expressed as mean ± s.d. (C and D) Representative pictures (C) and quantification (D) from Hoechst33342 and PI double-staining assay of HK-2 cells exposed to 50 ng/mL LPS with or without hypoxia in the presence of DAPK1 shRNA (sh.DAPK1) transfection or DAPK1 shRNA plus V5-tagged DAPK1 cotransfection. Experiments were performed three times and data are expressed as mean ± s.d. *, $P < 0.05$, one-way ANOVA, post hoc comparisons, Tukey's test. (E) Western blotting analyses assessing abundance of DAPK1 Ser308 phosphorylation in the LPS-stimulated cells with hypoxia followed by withdrawal for the indicated times. (F and G) Representative pictures (F) and quantification (G) from Hoechst33342 and PI

double-staining assay of HK-2 cells exposed to 50 ng/mL LPS with hypoxia followed by withdrawal for the indicated times. Experiments were performed three times and data are expressed as mean ± s.d.

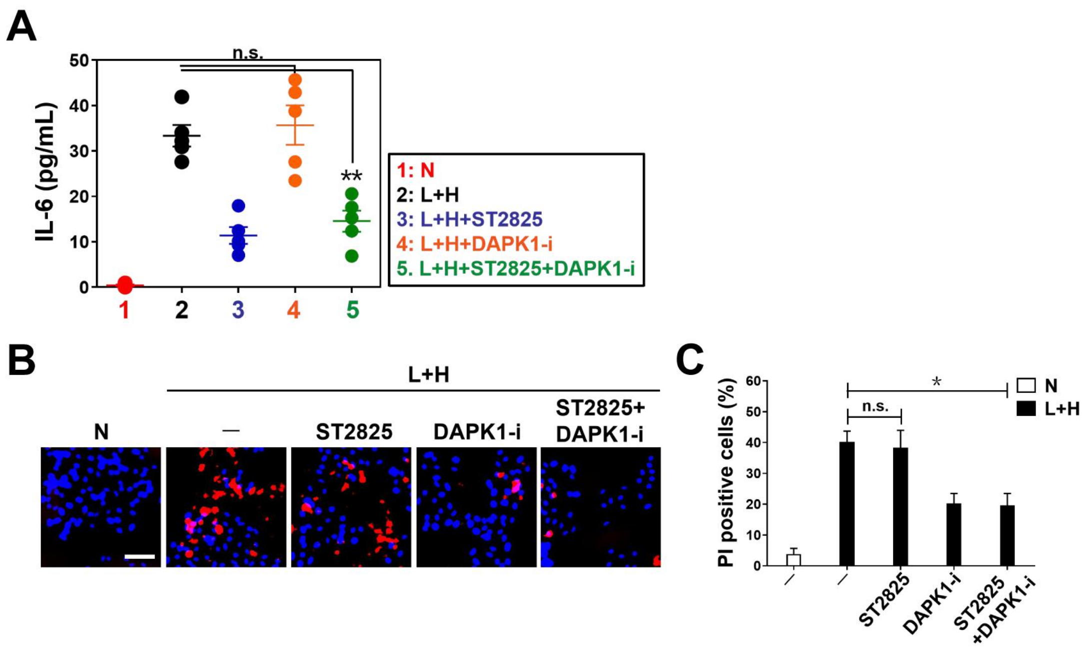

Supplemental Figure S5. The effects of MyD88 blockade in combination with DAPK1 deactivation in LPS plus hypoxia-costimulated tubular cells *in vitro*. (A) ELISA assay for IL-6 secretion from LPS plus hypoxia-costimulated HK-2 cells with 10 μM ST2825 and/or 10 μM DAPK1 inhibitor (DAPK1-i) treatment. Data are expressed as mean ± s.d. of at least three experiments. **, P<0.01, one-way ANOVA, post hoc comparisons, Tukey's test. n.s., no significant. (B and C) Representative pictures (B) and quantification (C) from Hoechst33342 and PI double-staining assay of LPS plus hypoxia-costimulated HK-2 cells with 10 μM ST2825 and/or 10 μM DAPK1 inhibitor (DAPK1-i) treatment. Experiments were performed three times and data are expressed as mean ± s.d. *, P<0.05, one-way ANOVA, post hoc comparisons, Tukey's test. n.s., no significant.

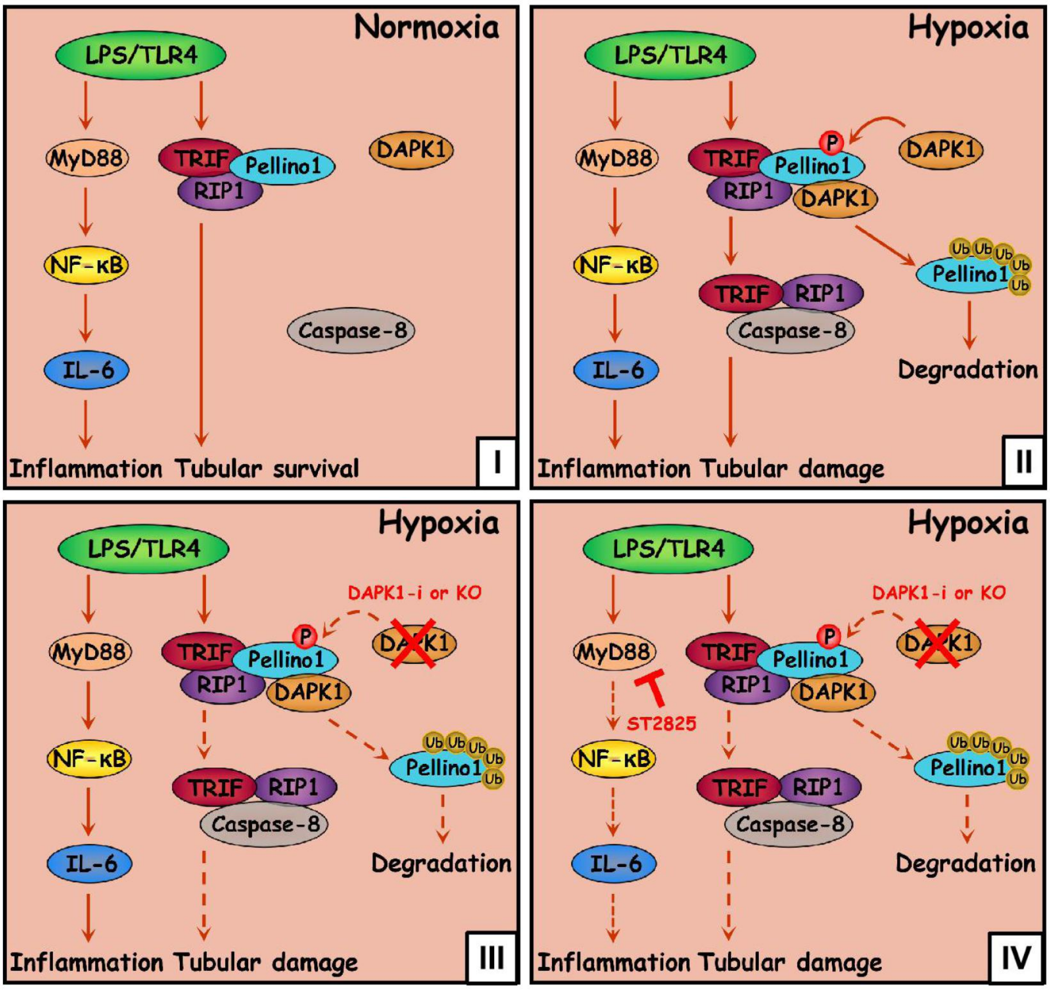

Supplemental Figure S6. Schematic models depicting the dominant role of DAPK1 in inflammation-coupling tubular damage initiated by LPS under hypoxia. Hypoxia activates DAPK1 to phosphorylate Pellino1 at Ser39, which leads to polyubiquitylation and turnover of Pellino1, followed by caspase-8 recruitment of TRIF-RIP1 signalosome and tubular damage in the case of LPS stimuli. Targeting DAPK1 and MyD88 synergistically prevents the inflammation-coupling tubular damage during septic AKI.
